# PARP1 promote autophagy in cardiomyocytes via modulating FoxO3a transcription

**DOI:** 10.1038/s41419-018-1108-6

**Published:** 2018-10-15

**Authors:** Cheng Wang, Wenjing Xu, Yanqing Zhang, Fengxiao Zhang, Kai Huang

**Affiliations:** 1 Clinical Center for Human Genomic Research, Union Hospital, Tongji Medical College, Huazhong University of Science and Technology, Wuhan, China; 2 Department of Cardiovascular Diseases, Union Hospital, Tongji Medical College, Huazhong University of Science and Technology, Wuhan, China

## Abstract

Autophagy is a key regulatory process in maintaining cellular homoeostasis via lysosome degradation. Growing evidence reveals that poly(ADP-ribose) polymerase-1 (PARP1) is involved in the progression of many cardiovascular diseases. This study was undertaken to discuss the role of PARP1 in cardiomyocyte autophagy. Our results demonstrated that PARP1 was activated in response to starvation-induced myocardial autophagy. We identified Forkhead box O (FoxO)3a as a substrate of PARP1. Upon PARP1 activation, poly(ADP-ribosyl)ation dissociated histone H1 from FoxO3a target gene promoter and promoted FoxO3a nuclear accumulation and binding activity to the target promoters, resulting in increased expression of autophagy related genes. Activated autophagy by PARP1 impaired mitochondrial metabolism and promoted cardiomyocyte death. And PARP1 silencing or specific inhibitors alleviated the promotion of FoxO3 activity upon starvation or myocardial ischemia, thus suppressing cardiac apoptosis and fibrosis. Together, these data indicate that PARP1-mediated poly(ADP-ribosyl)ation of FoxO3a plays a key role in cardiomyocyte autophagy. The utilization of PARP1 as a therapeutic target for related cardiovascular diseases would be desirable.

## Introduction

Autophagy is an evolutionally conserved mechanism for the turnover of cellular components and organelles via lysosomal degradation^1,2^. It occurs in karyocytes to serves an adaptive role to protect organisms against diverse pathologies. Cardiac myocytes have long been considered as terminally-differentiated cells that cannot be replaced. In response to stress, activated autophagy is responsible for the degradation of various macromolecules and organelles including mitochondria, which promotes heart cell survival^2,3^. However, the autophagic machinery triggered by severe stress could lead to self-destruction, and ultimately result in heart failure^3^.

Forkhead box O (FoxO) subfamily of transcription factors is characterized by a highly conserved Forkhead domain and comprise four members: FoxO1, FoxO3 (FoxO3a), FoxO4 and FoxO6^4^. FoxOs regulate various pathophysiological processes, including cellular atrophy, cell cycle, cell proliferation, and metabolism^4–6^. Moreover, it is well documented that FoxO3a is a positive modifier of autophagy^7^. The transcriptional activity of FoxO3a is conditioned by post-translational modifications such as phosphorylation, acetylation, ubiquitination, and methylation^8–10^.

As a post-translational modification enzyme, poly(ADP-ribose) polymerase-1 (PARP-1) is capable of stimulating or inhibiting the transcription during development and in response to environmental stimuli^11–13^. PARP-1 is historically known for its function to attach the ADP-ribose polymer chains to target proteins, a process known as poly(ADP-ribosyl)ation, and facilitates the process of DNA repair. However, increasing evidences suggest that PARP1 could be activated by oxidative stress or other stresses and PARP1-mediated poly(ADP-ribosyl)ation is involved in a wide range of biological processes^14^. Although we and others have recently identified poly(ADP-ribosyl)ation is related to several cardiovascular diseases, such as hypertension, various forms of heart failure or cardiomyopathies, circulatory shock, cardiovascular aging, myocardial hypertrophy, and atherosclerosis^15–18^, the underlying mechanism still remains unclear. And there is no evidence for PARP1 and autophagy in cardiac starvation or fraction, we then expected PARP1-mediated poly(ADP-ribosyl)ation is concerned with the progression of autophagy and cardiovascular diseases.

In the present study, we investigated the role of PARP1 in the autophagy of cardiomyocytes. Gain and loss of function studies revealed that activated PARP1 promoted autophagy in vivo and in vitro. Upon activation, PARP1-mediated poly(ADP-ribosyl)ation dissociated histone H1, then promoted FoxO3a transactivation, leading to increased autophagy related-gene expression. Furthermore, we demonstrated here inhibition of PARP1 could protect against cardiac ischemia injury by repressing autophagy via mediating FoxO3a signaling. Our results provide new insights into the functions of PARP1-dependent autophagy in cardiac functions.

## Materials and methods

### Primary culture of neonatal rat ventricular cardiomyocytes and reagents

Primary cultures of ventricular cardiomyocytes were prepared from 1-day-old Wistar rats as described^19^ using collagen II (CLS004176, Worthington) and trypsin (E7885, Sigma-Aldrich). A cardiomyocyte-rich fraction was obtained by centrifugation through a discontinuous Percoll gradient (P4937, Sigma-Aldrich). Cells were cultured in Dulbecco’s modified Eagle’s medium with 10% fetal bovine serum (GIBICO, 16000044). For glucose deprivation experiments, Hank’s buffered salt solution from GIBCO was used. The shRNA for Foxo3a (sc-37888), Histone H1(sc-62461), PARP1(sc-29438) were from Santa Cruz Biotechnology. PJ34(P4365), 3AB(A0788) and Bafilomycin A1(B1793) were from Millipore-Sigma,

### Western blot assay

Western blot assay was performed as previously described^20^. Tissue or cell extracts were prepared using a dounce homogenizer in cold RIPA buffer supplemented with cocktail. The homogenates were centrifuged at 12,000 rpm for 15 min, and the protein concentrations were determined using a protein assay from Thermo. The protein lysates were separated by SDS-PAGE and electrotransferred onto a nitrocellulose membrane. The membrane was scanned using the Image Lab statistical software (Bio-Rad). Antibodies against PAR (Trevigen, 4335-MC-100), PARP1 (Trevigen, 4338-MC-50), p-γH2AX (Ser139) (Trevigen, 4418-APC-020), Gabarapl1 (Abcam, ab86497), ATG12 (Abcam, ab109491), P62 (Cell signaling Technology, 39749), LC3 (Cell Signaling Technology CST, 3868), p-P53 (Ser15) (Cell signaling technology, 9284), p-ATM (Ser1981) (Thermo Fisher MA1-2020),FoxO3a (Cell signaling technology, 12829), pFoxO3a (Cell signaling technology, 9466), β-actin (Cell signaling technology, 3700), PGC1α (Abcam, ab54481), TFAM (Abcam, ab131607), NRF1 (Abcam, ab175932), Lamin B1 (Cell signaling technology,13435), α-tubulin (Cell signaling technology,3873) were used as primary antibodies.

### PARP1 activity assay

PARP1 activity was assayed using the universal colorimetric PARP assay kit (Trevigen, 4677-096-K), based on the incorporation of biotinylated ADP-ribose onto histone proteins. Cell lysates containing 50 μg of proteins were loaded into a 96-well plate coated with histones and biotinylated poly(ADP-ribose), allowed to incubate for 1 h, treated with strep-HRP, and read at 450 nm in a spectrophotometer.

### Visualization of autophagic vacuoles

Autophagy was measured by quantitation of GFP-LC3 puncta using fluorescence microscopy. Cells were infected with appropriate amounts of lentivirus carrying GFP-LC3 to express the close-to-endogenous level of GFP-LC3. After treatment, cells were fixed with 4% paraformaldehyde for 20 min and rinsed with PBS twice. Cells were mounted and visualized under a confocal microscope (Olympus FV-1,000).

### Transmission electron microscopy

Cells were fixed in 0.1 M sodium phosphate buffer (pH 7.4, PBS; Sigma, D5652–10 L) containing 4% formaldehyde (Fisher Chemical, F79) plus 1% glutaraldehyde (Fisher Chemical, O2957) for 4 h at 4 °C. After collection, samples were sent to the Wuhan Institute of Virology, Chinese Academy Of Sciences for detection. Autophagosomes are defined as double layer membrane structure containing cytoplasmic contents (mitochondria, lipid, damaged organelles, etc.) waiting to be degraded. Results were observed in a transmission electron microscope (FEI, Tecnai G20 TWIN, USA)

### Immunoprecipitation

After treatments, extracted proteins were incubated with primary antibody (anti-FoxO3a or PARP1) and rotated overnight at 4 °C. Magnetic beads were added for another 4 h. Bead complexes were washed with RIPA lysis buffer. Immunoprecipitates were then mixed with SDS loading buffer, boiled for 10 min and subjected to Western blots.

### Chromatin immunoprecipitation (ChIP)

The ChIP experiments were performed using the  EZ-Magna ChIP A/G Chromatin Immunoprecipitation Kit (Millipore,17-10086) according to the instructions provided by the manufacturer. Cells were sonicated, and the lysates were immunoprecipitated using an anti-FoxO3a antibody. In re-ChIP assays, chromatin was first immunoprecipitated with an anti-FoxO3a antibody, then eluted with 100 μl of elution buffer with 10 mM DTT at 37 °C for 30 min, diluted (25-fold) with dilution buffer (20 mM Tris-HCl [pH 8.0], 150 mM NaCl, 2 mM EDTA, 1% Triton X-100), and finally reimmunoprecipitated with IgG or an antibody against PARP1, PAR. Real-time PCR was performed using 1 μg of template DNA with specific primers. PCR products were separated on an ethidium bromide-stained 2% agarose gel.

### Luciferase assay

Cells were seeded in 24-well plates 1 day before transfection. For reporter assays, the cells were transiently co-transfected with reporter plasmid (WT-LC3 or MUT-LC3) in the presence of indicated treatment using Lipofectamine 2000. Firefly and Renilla luciferase activities were measured consecutively by using Dual-Luciferase® Reporter Assay System (Promega, E1910) according to the manufacturer’s instructions.

### Generation of recombinant adenovirus

The adenovirus kit, AdMax™ (Microbix), was used to generate adenovirus-based constructs according to the manufacturer’s recommendations. Briefly, the recombinant shuttle plasmids were co-transfected with the genomic plasmid into HEK293 cells to produce the recombinant viral particles, and viral titers were enriched by two rounds of infection in HEK293 cells. To generate adenovirus encoding full-length mouse ATG12 (Pubmed No. NM_026217.3). ATG12 cDNA fragments were transferred from pcDNA3-based vectors to the shuttle plasmid pDC316. The CMV-null adenovirus was used as the negative control (Ad-Null).

### Quantitative reverse transcription-polymerase chain reaction

Total RNA was extracted by use of TRIzol (Invitrogen, 15596026). For mRNA quantification, 2 μg RNA was reverse-transcribed into cDNA using a PrimeScript™RT reagent Kit (Takara, RR037), followed by quantitative real-time PCR (qPCR) with SYBR Green (Bio-Rad, 1725270) and a Bio-Rad CFX-96 real-time system.

### Flow cytometry

Living transfectants were stained with 100 nM MitoTracker Green (Molecular Probes) for 30 min at 37 °C, treated with trypsin and subjected to a flow cytometric analysis with an Epics Elite ESP (Coulter).

### Nuclear/cytosolic isolation

The cells were washed twice with ice-cold phosphate-buffered saline and then scraped from plates in 100 ml of ice-cold lysis buffer [20 mM HEPES, pH 7.4, 10 mM NaCl, 1.5 mM MgCl_2_, 20% glycerol, 0.1% Triton X-100, 1 mM dithiothreitol, and protease inhibitor mixture (Sigma, S8820)]. The cell lysate was centrifuged at 1000 rpm for 1 min at 4 °C. The supernatant contained the cytosolic fraction. The nuclear pellet was resuspended in 50 ml of lysis buffer and 8.3 ml of 5 M NaCl was added to lyse the nuclei. This mixture was rotated for 1 h and then centrifuged at 15,000 rpm for 15 min at 4 °C. The supernatant contained the soluble nuclear fraction and the pellet was re-suspended in 30 ml of lysis buffer. An equal volume of SDS-PAGE loading buffer was added to each fraction.

### Immunofluorescence staining

After cells were placed on glass slides at an appropriate density, they were fixed with 4% paraformaldehyde for 15 min, were permeabilized with 0.1% Triton in PBS for 30 min at room temperature, and were incubated overnight in blocking solution containing antibody against FoxO3a. Cy3-conjugated secondary antibody (Proteintech, SA00009-2) was used for detection. The nucleus was stained with DAPI, and sections were then viewed under the fluorescence microscope (Olympus, Japan).

### Oxygen consumption

Cells were plated on 60 mm Petri dishes and treated according each experiment. Cells were then trypsinized, and the suspension (in PBS) was placed in a chamber, coupled to a Clark electrode 5331 (Yellow Springs Instruments) where the oxygen uptake was measured polarographically.

### Measurement of intracellular ATP

ATP was measured using the ATP bioluminescence assay kit HS II (Roche Applied Science 11699709001,). Briefly, cells were incubated with different concentrations of glucose (1–10 mM) and sodium oxamate (5–100 mM) as indicated. After 2 h, intracellular ATP content was measured following the manufacturer’s instructions. Luminescence intensity was normalized to the protein present in each sample.

### Cell viability

Cell viability was assessed by a MTT assay, as directed by the manufacturer (Invitrogen, A13261). The integrity of the plasma membrane was assessed by determining the ability of cells to exclude PI/Annexin V. The level of PI/Annexin V incorporation was quantified in a FACScan flow cytometer.

### Animal protocols

Eight-to ten-week-old male C57/BL6J mice were used for assessment of 7 days ligation of anterior descending branch (LAD) as described previously^21^. Sham-operated control animals were treated in an identical manner except that the LAD was not ligated. All procedures were approved by the Animal Care and Utilization Committee of Huazhong University of Science and Technology (project number 2016S745).

### Histological analysis

Hearts were fixed in 4% paraformaldehyde overnight and then embedded in paraffin. Paraffin blocks were sliced into sections of 6 μm in thickness. The collagen content was determined by Masson staining. To detect apoptosis, tissue sections were used for Cell Light EdUTP TUNEL In situ Detection Kit (RIBOBIO, R11057). TUNEL-positive cells were imaged under a fluorescence microscope

### Statistical analysis

Values are shown as the means ± s.e.m. of at least three independent experiments. Homogeneity of the variance was assessed by the *F* test (two groups) or Brown-Forsythe test (≥3 groups). The statistical significance of differences between two groups was analyzed by Student’s *t*-tests. For comparing more than two means, one-way analysis of variance with the Newman-Keuls post-hoc analysis was employed. Values of *P* < 0.05 were considered statistically significant. All statistical analyses were performed using SPSS software (version 22.0, SPSS Inc).

## Results

### PARP1 mediates starvation induction of autophagy in cultured cardiomyocytes

**Fig. 1:**
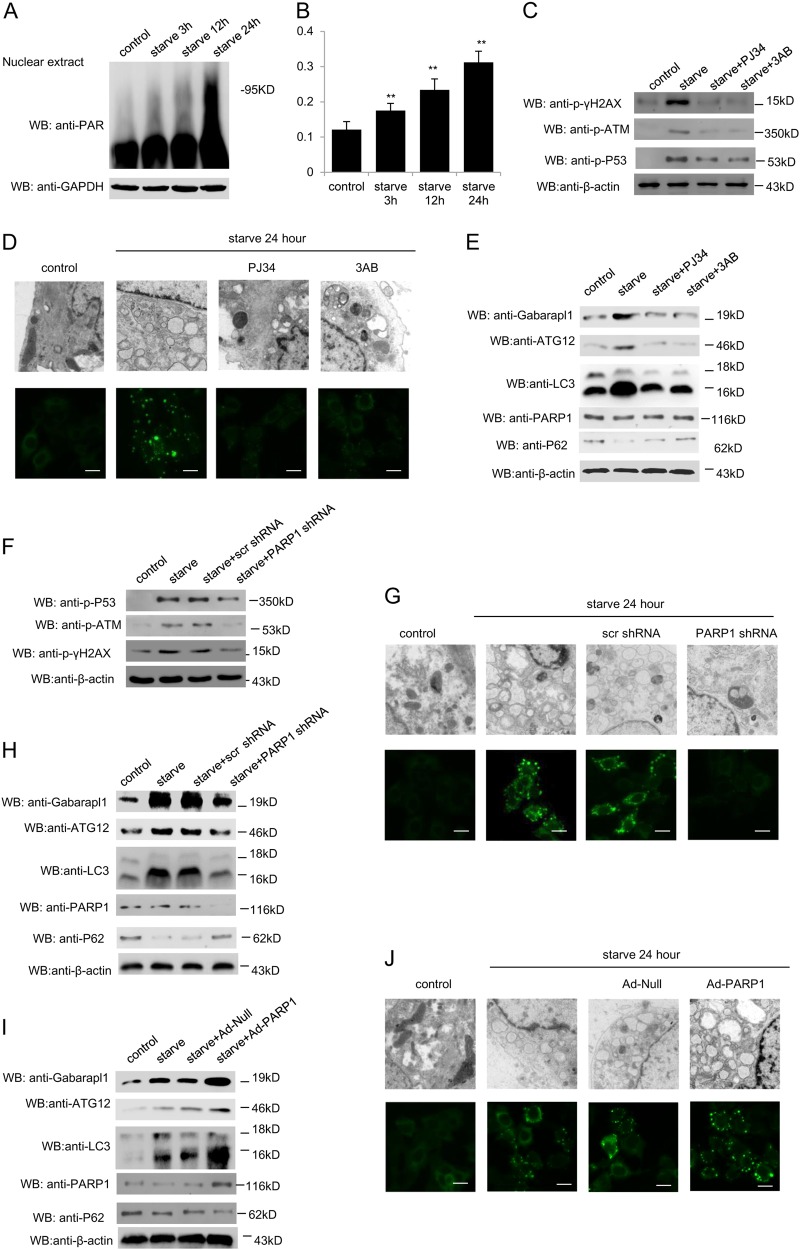
PARP1 mediates starvation induction of autophagy in cultured cardiomyocytes. Primary neonatal rat cardiomyocytes were starved for the indicated different times. **a** The protein levels of total poly(ADP-ribosyl)ation were detected by western blot analysis. **b** Cellular PARP activity was assayed as described in Materials and Methods. *N* = 8 for each group. ^**^P < 0.01 vs. control. **c**, **j** Primary rat cardiomyocytes were treated either with PJ34 (10 μM) or 3AB (10 mM), or infected with PARP1 shRNA or an scramble (Scr) shRNA for 24 h, or infected with Ad-PARP1 or Ad-Null and then starved for 24 h. The protein level of DNA damage marker (p-γ-H2AX, p-ATM, p-p53) (**c** and **f**), autophagy marker (Gabarapl1, ATG12, LC3), PARP1 and p62 (**e**, **h**, and **i**) were detected by western blot analysis. Representative electron microscopy and GFP-LC3 fluorescence confocal images are displayed to assess the effect on starvation-induced autophagy (**d**, **g**, and **j**)

Starvation or nutrient deprivation is a physiological cellular stress to induce autophagy. Primary rat cardiomyocytes were subjected to glucose deprivation experiments. TEM (transmission electron microscopy) analysis confirmed autophagy-derived ultrastructural changes include concentrical membrane structures engulfed in autophagosomes and ER dilation after starvation (Fig.S2A). DNA damage as an early event of starvation-induced autophagy was measured by western blot with anti-phospho-Histone γ-H2AX antibody (Fig. S2B). To study the role of poly(ADP-ribosyl)ation in starvation-induced autophagy, starved cardiomyocytes were subjected to PARP activity assay and western blot assay with anti-poly(ADP-ribose) polymer (PAR) antibody. Results indicated that PARP activity was dramatically increased with time in starved myocardial cells (Fig. 1a, b). PARP inhibitor 3AB (3-aminobenzamide) or PJ34 (9N-(6-oxo-5,6-dihydrophenanthridin-2-yl)-N,N-dimethylacetamide) was added in cardiomyocytes. Phosphorylation of γ-H2AX, p53 and ataxia telangiectasia mutated (ATM) was measured to explore the DNA damage. Western blot assay revealed that PARP inhibitors dramatically ameliorated starvation-induced DNA damage (Fig. 1c). Autophagosomes were then monitored by TEM observation and GFP-Light Chain 3(LC3) fluorescence analysis. Both 3AB and PJ34 could attenuate autophagosome formation (Fig. 1d). Accordingly, as shown in Fig. 1e, starvation promoted the transcriptional expressions of several autophagy-related genes, including Gabarapl1, autophagy-related protein 12 (ATG12) and LC3, while treatment with PARP inhibitors consistently inhibited these gene expressions (Fig. 1e, S3A and S4A). As an autophagy substrate, the expression of p62 is negative correlated with autophagy activity. Western blot assay also proved that 3AB and PJ34 could increased the expression of p62 (Fig. 1e).

To examine whether it was PARP1 that mediated the effects of poly(ADP-ribosyl)ation in starved induced-autophagy, cells were infected with lentivirus encoding short hairpin (sh) RNA against PARP1 (shPARP1) to specifically knock down PARP1. Consistent with PARP inhibitor, cells with PARP1 shRNA showed less DNA damage, decreased autophagy and related gene expressions, promoted the p62 expression (Fig. 1f–h, S3B and S4B). To further confirm that PARP1 knockdown lead to impaired autophagy in cardiomyocytes, gain-of-function assays were performed by over-expression of PARP1. Cells were infected with adenovirus encoding human PARP1 gene (Ad-PARP1) or empty vector. Results showed that infection with Ad-PARP1 resulted in up-regulation of Gabarapl1, ATG12 and LC3 (Fig. 1i, S3C and S4C). Moreover, infection with Ad-PARP1 vs. empty vectors increased autophagosome accumulation (Fig. 1j). These results support the conclusion that PARP1 has a major role in the transcriptional control of the autophagy program in cardiomyocyte and raise the question of which downstream pathways mediate the effect of PARP1.

### PARP1 is required for starvation to increase Fox3a transactivation

**Fig. 2:**
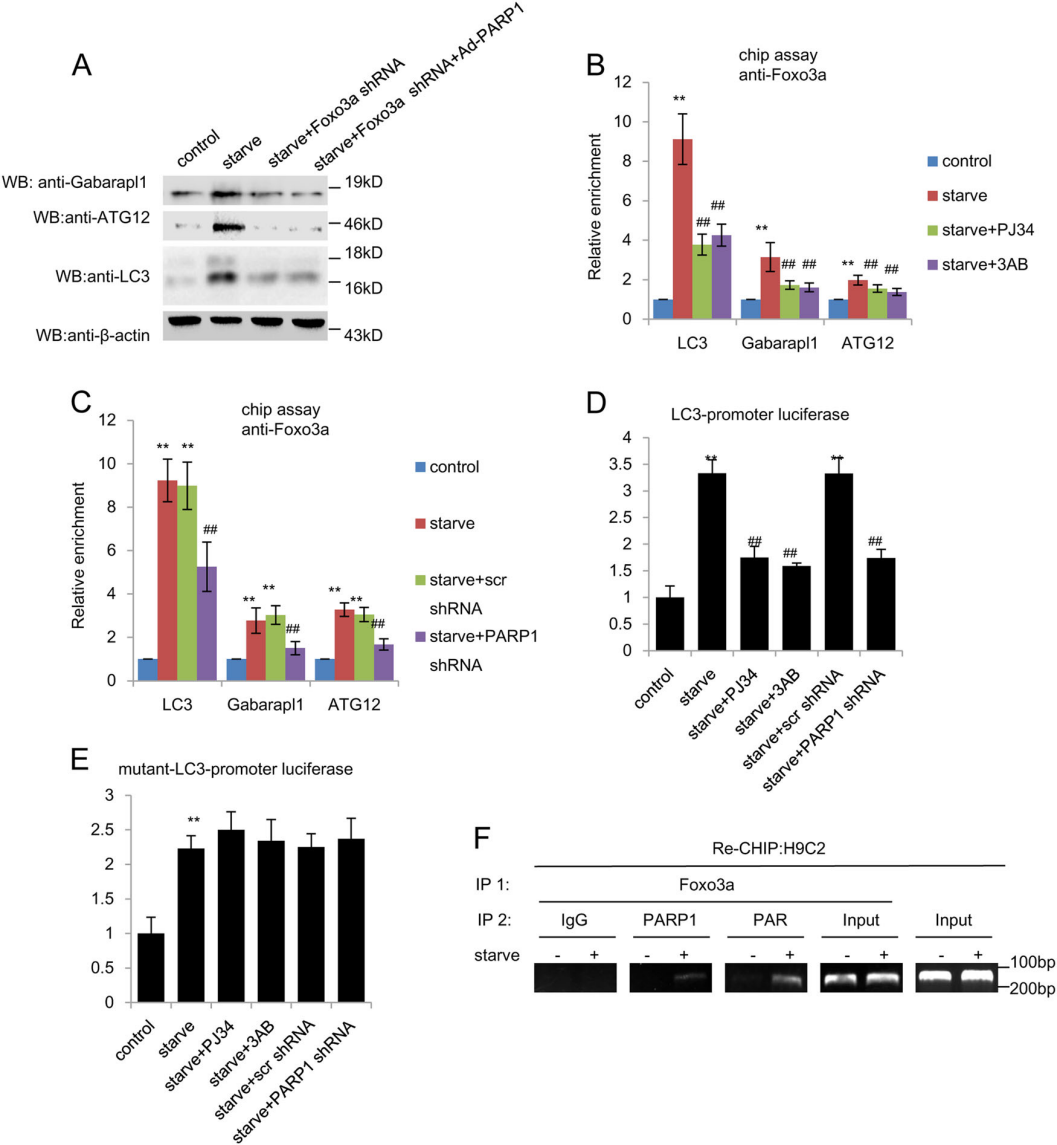
PARP1 is required for starvation to increase Fox3a transactivation. **a** Primary neonatal rat cardiomyocytes were infected with FoxO3a shRNA or FoxO3a shRNA + Ad-PARP1 adenovirus for 24 h and were then exposed to starvation for 24 h. The protein level of Gabarapl1, ATG12 and LC3 were detected by western blot analysis. **b** and **c** ChIP-PCR assays using an anti-FoxO3a antibody for amplification of LC3 promoters in cardiomyocytes treated with 10 μM PJ34 or 10 mM 3AB for 24 h or transfected with PARP1 shRNA or an unrelated shRNA for 24 h, with or without starvation treatment (24 h). *N* = 5 for each group. ^**^P < 0.01 vs. control; ^##^*P* < 0.01 vs. starve or starve +  Scr shRNA. **d** and **e** Relative LC3 (WT-LC3) or mutant (mut-LC3) luciferase reporter activity in primary rat cardiomyocytes treated as indicated. *N* = 8 for each group. ^**^P < 0.01 vs. control; ^##^*P* < 0.01 vs. starve or starve +  Scr shRNA. **f** In re-ChIP assays, chromatin was first immunoprecipitated with an anti-FoxO3a antibody and was then re-immunoprecipitated with an anti-PAR antibody, an anti-PARP1 antibody. IgG served as a negative control

It is well documented that FoxO3a is necessary and sufficient for the induction of autophagy in vivo, leading us to wonder whether there exists some cooperation between PARP1 and FoxO3a. Our results proved that PARP1 could directly bind to and poly(ADP-ribosyl)ate FoxO3a in cardiomyocytes, which was consistent with a latter research (Fig. S5). Moreover, FoxO3a knockdown abolished PARP1 overexpression-induced the expressions of FoxO3a target genes (Fig. 2a, S3D, S4D), indicating that PARP1 may mediate starvation-induced cardiomyocyte autophagy by regulating FoxO3a signaling pathway.

FoxO3a is directly involved in the transcriptional regulation of autophagy-related genes and mainly focuses on LC3, Gabarapl1 and ATG12^22^. ChIP assay with FoxO3a antibody was performed to determine whether poly(ADP-ribosyl)ation would affect the binding activity of FoxO3a to its target gene promoter. Results showed that PARP inhibitors decreased the starvation-induced recruitment of FoxO3a to the promoter of autophagy related genes (LC3, Gabarapl1 and ATG12) in cardiomyocytes (Fig. 2b). In support of this, PARP1 knockdown exhibited less recruitment of FoxO3a to promoter of above autophagy-related genes than scramble shRNA (Fig. 2c).

To determine the effects of PARP1 activation on FoxO3 transcriptional activity, the region of the LC3 promoter containing the proximal FoxO site (WT-LC3) or mutant LC3 (MUT-LC3) promoter was cloned into luciferase reporter vector and transfected into myocardial cells, respectively. After fasting, treatment with shPARP1 or PARP inhibitor displayed a much lower luciferase activity, indicating that inhibition of PARP1 decreased the WT-LC3 promoter activity (Fig. 2d). Furthermore, neither shPARP1 nor PARP inhibitor treatment could affect the luciferase activity driven by MUT-LC3 promoter (Fig. 2e). In line with this, Re-ChIP (ChIP on ChIP) showed that PARP1 and poly(ADP-ribosy)lated proteins, including poly(ADP-ribosy)lated FoxO3a, could recruit to the promoter of LC3 (Fig. 2f). Taken together, PARP1 mediated poly(ADP-ribosy)lation promoted FoxO3a recruitment to its target gene promoter and strengthened the transcriptional activity.

### PARP1 mediates nuclear localization of FoxO3a

**Fig. 3:**
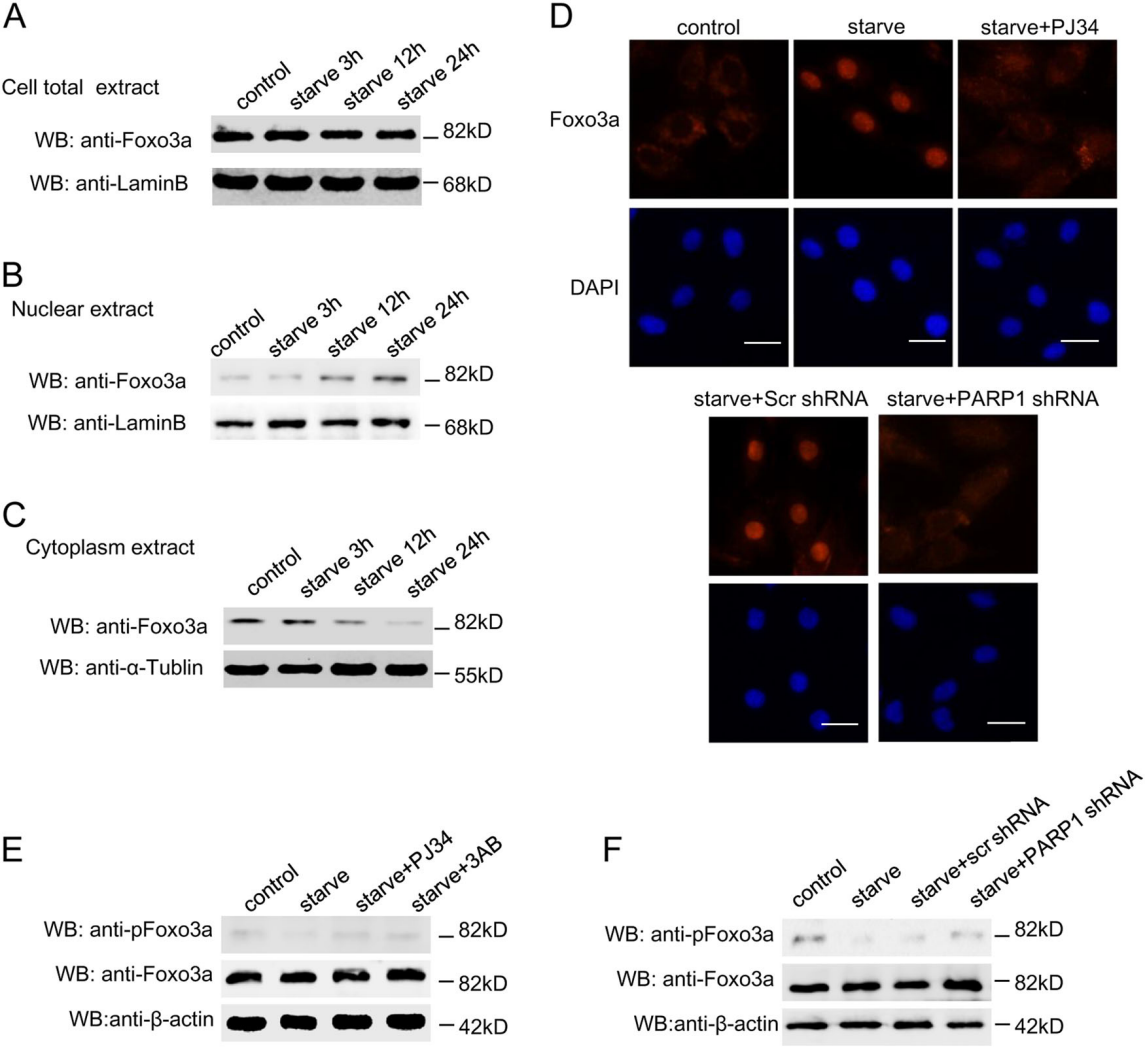
PARP1 mediates nuclear localization of FoxO3a. **a**–**c** Primary neonatal rat cardiomyocytes were starved for indicated different times. Western-blot assay was used to detect the expression of FoxO3a in total extracts (**a**), nuclear extracts (**b**) or cytoplasm extracts (**c**), respectively. Primary rat cardiomyocytes were pre-treated with 3AB (10 mM,) or PJ34 (10 µM), or transfected with PARP1 shRNA or an unrelated shRNA for 24 h followed by starvation (24 h). **d** Confocal immunofluorescence assay was used to detect the expression of FoxO3a (red fluorescence), Hoechst (blue fluorescence) was used to stain the cell nuclei (scale bar = 40 µm). **e** and **f** Western-blot assay was used to detect the expression of total FoxO3a and pFoxO3a

The transcription activities of FoxOs are regulated by a broad variety of stimuli which control FoxO protein expression and subcellular localization. Sequestration of FoxO proteins in the cytoplasm prevents FoxO-dependent gene regulation. In this study, the total content of FoxO3a was almost invariable after starvation (Fig. 3a). But western blot assay showed that expression of FoxO3a was dramatically increased with time in nuclear extracts from starved myocardial cells, and simultaneously reduced in cytoplasm extracts (Fig. 3b, c). When treated with PJ34 or PARP1 shRNA, the nuclear FoxO3a content was comparably decreased and then confined to the cytoplasm, as revealed by confocal immunofluorescence assay (Fig. 3d), suggesting PARP1-dependent poly(ADP-ribosy)lation could affect FoxO3a localization.

Phosphorylation has been involved in the subcellular location and transcriptional regulation of FoxO. We then explored the role of serum starvation in phosphorylation of FoxO3a. As shown in Fig. 3e, f, starvation inhibited phosphorylation of FoxO3a. We further explored the possible participation of PARP1 in regulating cardiac FoxO3a. Western blot assay showed that the delivery of PARP inhibitor or shPARP1 reversed starvation-induced de-phosphorylation of FoxO3a (Fig. 3e, f), which was in accordance with the decreased nuclear accumulation (Fig. 3d). Therefore, it appears that PARP1 promotes de-phosphorylation of FoxO3a and induce its nuclear import in cardiomyocytes.

### PARP1 promotes FoxO3a activation by excluding histone H1 from its target promoter

**Fig. 4:**
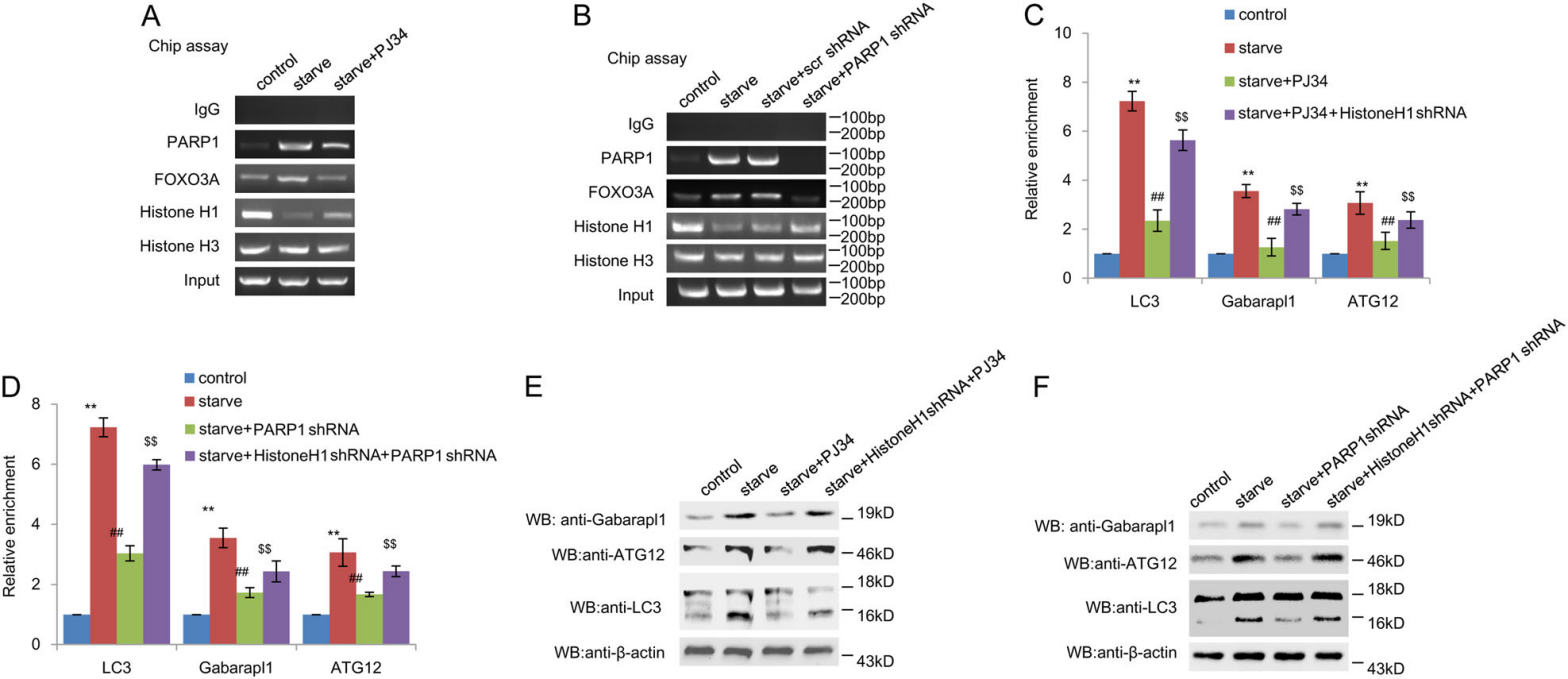
PARP1 promotes FoxO3a activation by excluding histone H1 from its target promoter. **a** and **b** Primary neonatal rat cardiomyocytes were pre-treated with 3AB (10 mM,) or PJ34 (10 µM), or transfected with PARP1 shRNA or an unrelated shRNA for 24 h followed by starvation (24 h). Soluble chromatin was then prepared for ChIP assays with antibodies against the indicated proteins. After infection with Histone H1 shRNA for 24 h, cardiomyocytes were treated with the PARP1 shRNA or PJ34 and were then exposed to starvation for 24 h. **c** and **d** Soluble chromatin was then prepared for ChIP assays with antibodies against Foxo3a for amplification of LC3 promoters. *N* = 5 for each group. ^**^P < 0.01 vs. control; ^##^*P* < 0.01 vs. starve; ^$$^*P* < 0.01 vs. starve + PJ34 or starve + PARP1 shRNA. **e** and **f** Western blot assay was used to detect the expression of Gabarapl1, ATG12 and LC3 in different treated groups

In the nucleus, FoxO3a-binding sites are marked with the combination of histone modifications specific for enhancers^23^. Histone modifications could induce recruitment of FoxO3a to its binding sites in target gene promoter to facilitate the transcription activity of FoxO3a^23^. We then explored the possible participation of histone proteins in regulation of FoxO3a by PARP1. Chip assays indicated that, in contrast to FoxO3a/LC3 promoter, starvation inhibited the formation of histone H1/LC3 promoter, not histone H3 (Fig. 4a, b). Whereas treatment with PARP1 inhibitor PJ34 or PARP1 shRNA restored the binding of histone H1 to FoxO3a target gene promoter (Fig. 4a, b).

In nucleus, histone H1 and PARP1 exhibit a reciprocal pattern of chromatin binding at gene promoters, and PARP1 could exclude histone H1 from PARP-1-regulated promoters^24^. As the substrate of PARP1, poly(ADP-ribosyl)ation of histone H1 regulates the gene transcription via chromatin alterations^25^. The cultured myocardial cells were transfected with histone H1 shRNA and then were submitted to Chip analysis and real time PCR assay. As shown in Fig. 4c, d, PARP1 inhibitor PJ34 and PARP1 silencing decreased recruitment of FoxO3 to target gene promoter, but retarded by histone H1 knockdown. Consistently, histone H1 knockdown could reverse the decreased expressions of LC3, Gabarapl1 and ATG12 by PJ34 and PARP1 silencing (Figs. 4e, f, S3E and F, S4E and F).

Histone H1 is responsible for the deacetylation of FoxO3. Sirt1 which is a histone acetyltransferase (HAT), could deacetylate FoxO factors to regulate its function. The effects of Sirt1 on nuclear retention ability of FoxO3 and target gene expressions were also explored in the present study. Results showed that over-expression of Sirt1 promoted the nuclear accumulation of FoxO3 and increased its target gene expression (Fig.S6A and B). Primary rat cardiomyocytes were then co-transfected with Sirt1 and p300 siRNA. Compared with sirt1 siRNA treatment, co-transfection of Sirt1 and p300 siRNA facilitated the nuclear localization of FoxO3 and its target gene expression (Fig. S6C and D).

We also mutated major sites within FoxO3, acetylated lysine residues (K242, K259, K290, and K569), known to be regulated by HATs and HDACs. We then check whether these mutations affected the mediation of starvation-induced FoxO3 transcriptional activation. Chip assays indicated that, mutation of these sites almost did not change the recruitment of FoxO3a to the promoter of autophagy related genes (Fig. S6E). We next explored whether these mutations could affect the mediation of PARP1 on FoxO3. As shown in Figure S6F, FoxO3 mutation has no significant effect on poly(ADP-ribosyl)ation of FoxO3.These data suggested directed acetylation of FoxO3 cannot influence the regulation of PARP1 on autophagy related gene expression.

Thus, the present work demonstrates that PARP1 acts as an activator of FoxO3a by excluding histone H1 from the promoter of its target genes.

### PARP1 impaires mitochondrial metabolism through FoxO3a

Mitochondria are identified as the regulators of autophagy, loss of mitochondrial ATP production can thus induce autophagy^26^. To test whether PARP1 activation is associated with mitochondrial function, we measured basal oxygen consumption rates and ATP levels in vivo in cardiomyocytes. Results showed that the basic respiration and ATP contents were decreased in starved cardiomyocytes compared to control cells. However, PARP1 inhibitor PJ34 and PARP1 shRNA treatment restored the inhibition of basal oxygen consumption and ATP contents (Fig. S7 A,B,D and E). Furthermore, PARP1 inhibitor PJ34 and PARP1 shRNA treatment also reversed the inhibition of mitochondrial function related gene expression, including PGC1α, TFAM, and NRF1 (Fig. S8A and B). In contrast, overexpression of PARP1 aggravated the starvation-induced oxygen consumption and ATP contents (Fig.S7G and H). We also collected the confocal images of cardiomyocytes labeled with green-fluorescing MitoTracker Green (MTG) to visualize mitochondria. Results showed that neither PARP inhibition and PARP1 over-expression affected the starvation-induced physical loss of mitochondria (Fig. S7 C,F and I ).

To further examine the role of FoxO3a in mediating the effects of PARP1 on mitochondrial metabolism, FoxO3a was knocked down by shRNA in cardiomyocytes. As shown in Figure S7J, Ad-PARP1-induced oxygen consumption was abolished along with FoxO3a shRNA transduction. Consistently, the knockdown of FoxO3a also abrogated the decreased ATP synthase by Ad-PARP1 (Fig. S7K). Moreover, MTG also revealed that knockdown of FoxO3a could not reverse the starvation-induced mitochondrial deficiency (Fig. S7L). Taken together, FoxO3a was indispensable for the impacts of PARP1 on cardiac mitochondrial energy metabolism.

### PARP1-mediated autophagy promotes cardiomyocyte death through FoxO3a pathway

**Fig. 5:**
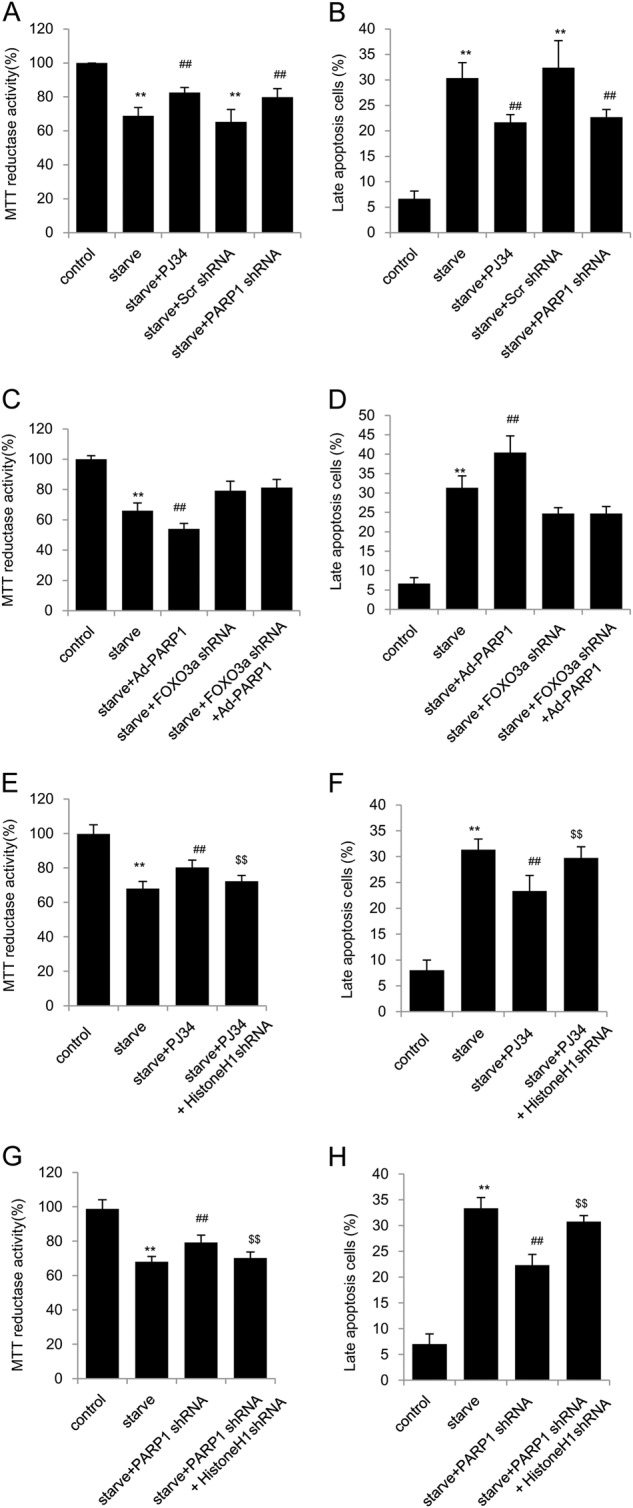
PARP1-mediated autophagy promotes cardiomyocyte death through FoxO3a pathway. Primary neonatal rat cardiomyocytes were treated with PJ34 (10 μM), or infected with PARP1 shRNA, and then starved for 24 h. **a** Cell viability was determined by MTT colorimetric assay. **b** Cells were labeled with PI/Annexin V and analysed by fluorocytometry. *N* = 5 for each group. ^**^P < 0.01 vs. control; ^##^*P* < 0.01 vs. starve or starve + scramble shRNA. **c** and **d** After infection with FoxO3a shRNA for 24 h, cardiomyocytes were infected with Ad-PARP1 for 24 h and were then exposed to starvation for 24 h. Cell viability (**c**) and PI/Annexin V-labeled apoptosis cells (**d**) were accessed. *N* = 5 for each group.^**^P < 0.01 vs. control; ^##^*P* < 0.01 vs. starve; ^$$^P < 0.01 vs. starve + Ad-PARP1. **e** and **h** cells were first infected with Histone H1 shRNA, and treated with PJ34 or PARP1 shRNA and then exposed to starvation for 24 h. Cell viability (**e** and **g**) and PI/Annexin V-labeled apoptosis cells (**f** and **h**) were accessed. *N* = 5 for each group. ^**^P < 0.01 vs. control; ^##^*P* < 0.01 vs. starve; ^$$^*P* < 0.01 vs. starve + PJ34 or starve+PARP1 shRNA

Autophagy shows both biological effects on cell death. It can not only block the induction of apoptosis by inhibiting the activation of apoptosis-associated caspase which could reduce cellular injury, but also help to induce apoptosis^27^. Viability of cardiomyocyte was monitored using the MTT reduction assay TUNEL and PI/Annexin V incorporation. Interestingly, we found starvation led to decreased cell viability. Starved primary rat cardiomyocytes were starved and treated with PJ34 for different times. The results showed that PJ34 treatment aggravated the cytotoxicity of starvation for 4 h, while inhibited late apoptosis of cells starved for 24 h (Fig. S9). These findings suggest that PARP1 might play distinct roles at different levels of autophagy. Furthermore, inhibition of PARP1 by PJ34 or shPARP1 relieved but ectopic PARP1 expression aggravated the cytotoxicity of starvation for 24 h (Fig. 5a–d), indicating a worsen effect of PARP1 related autophagy in cardiomyocyte viability.

To determine whether Histone H1-FoxO3a was essential for PARP1 activation stimulated cardiomyocyte cell death, myocardial cells were transfected with FoxO3a shRNA. Interestingly, the cytotoxicity of Ad-PARP1 was nearly hindered by shFoxO3a (Fig. 5c, d). Moreover, we selectively knocked down histone H1 in cardiomyocytes and performed MTT and PI/Annexin V assays. The sh-histone H1 treatment dramatically restrained the protection on cell viability from PARP1 inhibition PJ34 or PARP1 knockdown (Fig. 5e–h) Therefore, all these results suggest that, PARP1 may participate in excessive autophagy induced cell death through histone H1-FoxO3a-autophagy pathway.

### PARP1 inhibition attenuates autophagy to protect cardiac apoptosis and cardiac dysfunction induced by myocardial ischemia

**Fig. 6:**
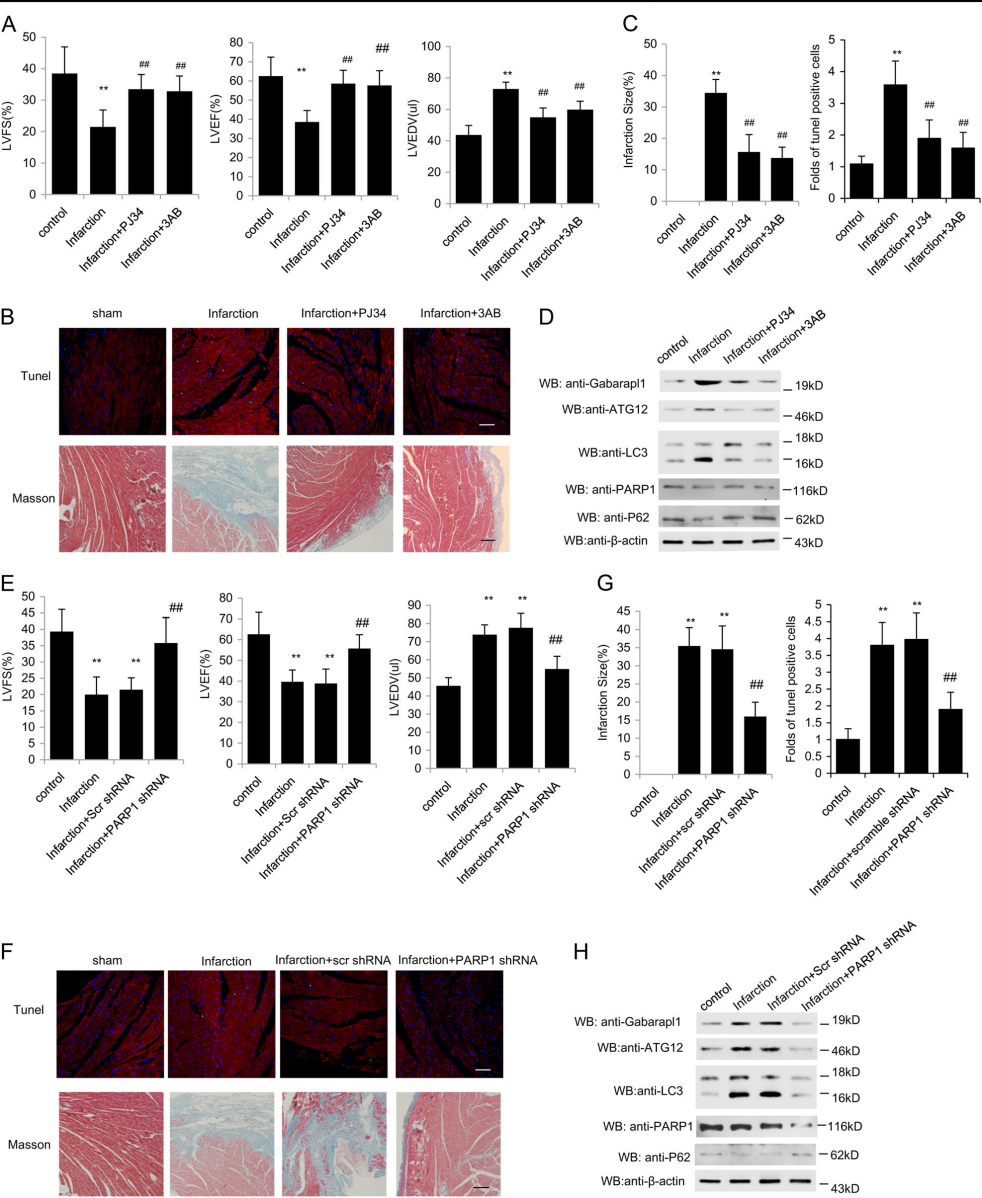
PARP1 inhibition attenuates cardiac apoptosis and cardiac dysfunction induced by myocardial ischemia via reducing autophagy After 8–10-week-old male C57/BL6J mice were subjected to LAD ligation, mice either were intraperitoneal injected with PJ34 (20 mg/kg/day) or 3AB (30 mg/kg/day) or intramuscular injected with PARP1 shRNA or an unrelated shRNA. **a** and **e** The quantification of LVFS %, LVEF %, and LVEDV (ul) were conducted. *N* = 5 for each group. ^**^P < 0.01 vs. control; ^##^*P* < 0.01 vs. Infarction. The representative TUNEL and Masson staining images were accessed (**b**, **c**, **f**, and **g**). Western-blot assay was used to detect the expression of Gabarapl1, ATG12, LC3, PARP1, and p62 in the areas proximal to infarct/ischemic zone in MI mice (**d** and **h**)

During the process of ischemia injury, the energetic status of cell is dynamically changed with nutrient deprivation^28^. Autophagy may serve to maintain energy production in response to energy deprivation, and also may be responsible for the clearance of long-lived proteins and dysfunctional organelles^29^. However, the excessive autophagy triggered by severe stress could lead to self-destruction, and ultimately result in cell death. We then investigated the effects of PARP1 and autophagy in mice myocardial infarction (MI). There were no significant differences in weight loss or survival among groups (Data no shown). To assess the actions of PARP1 on LV function post-MI, echocardiography measurements were performed. Echocardiography data reflected a reduced left ventricular fractional shortening (LVFS %) and left ventricular ejection fractions (LVEF %) and increased end-diastolic chamber size (LVEDD ul), at day 7 in MI group compared with no-MI control, indicating impaired LV dysfunction after ischemia injury (Fig. 6a). Both 3AB and PJ34 treatment improved the LVFS % and LVEF %, and decreased LVEDD compared to MI group (Fig. 6a).

Quantification of TUNEL staining in the infarcted apical region revealed that MI significantly enhanced myocardial apoptosis, and this effect was attenuated by PARP inhibitor treatment (Fig. 6b, c). Furthermore, western blot also showed that the PARP inhibitors treatment inhibited the MI-induced expressions of autophagy related genes LC3, Gabarapl1 and ATG12, and increased p62 expression, while the expression of PARP1 remained unchanged (Fig. 6d, S3G and S4G). In MI heart, death of cardiomyocytes are replaced with a permanent collagenous scar instead of new cardiac muscle tissue^30^. Masson trichrome staining were then used to assess infarct area and myocardial fibrosis. Masson Trichrome staining displayed overt myocardial fibrosis following MI, and PARP inhibitor treatment attenuated the MI-induced fibrosis (Fig. 6b, c). Meanwhile, expressions of fibrosis related gene, including TGFβ, CoLIA1 CoLIIA1 and TIMP, were restricted (Fig. S10A).

PARP1 was then specifically knocked down by infection of shPARP1 in MI mice. In accordance with PARP inhibitor, shPARP1 treatment enhanced LVFS and LVEF compared to MI group (Fig. 6e). Furthermore, PARP1 knockdown significantly attenuated the MI-induced apoptosis with consistent myocardial fibrosis and related gene expression (Fig. 6g, S10B). Accordingly, infection with shPARP1 decreased the autophagy and related gene expression (Gabarapl1, ATG12 and LC3) and raised the expression of p62 in the myocardium, as compared to scrambled shRNA (Scr shRNA) (Fig. 6h, S3H and S4H). These data suggest that PARP1 inhibition may indirectly benefit MI induced myocardial dysfunction by inhibiting cardiac autophagy.

### PARP1 inhibition protects the heart against ischemia by inhibition of autophagy initiation

To further confirm the change in autophagy related gene expression by autophagic initiation promotion or autophagic flux blocking, the autophagy inhibitor bafilomycin A1 (Baf A1) were used in starved myocardial cells to inhibit the fusion of autophagosomes and lysosomes. Western blot assay showed that, when Baf A1 was added, there was significant augmentation of autophagy related genes and P62, and PARP1 inhibitor 3AB or PARP1 knockdown could reversed this effect (Fig. S11). These suggested that PARP1 inhibition participated autophagy initiation.

**Fig. 7:**
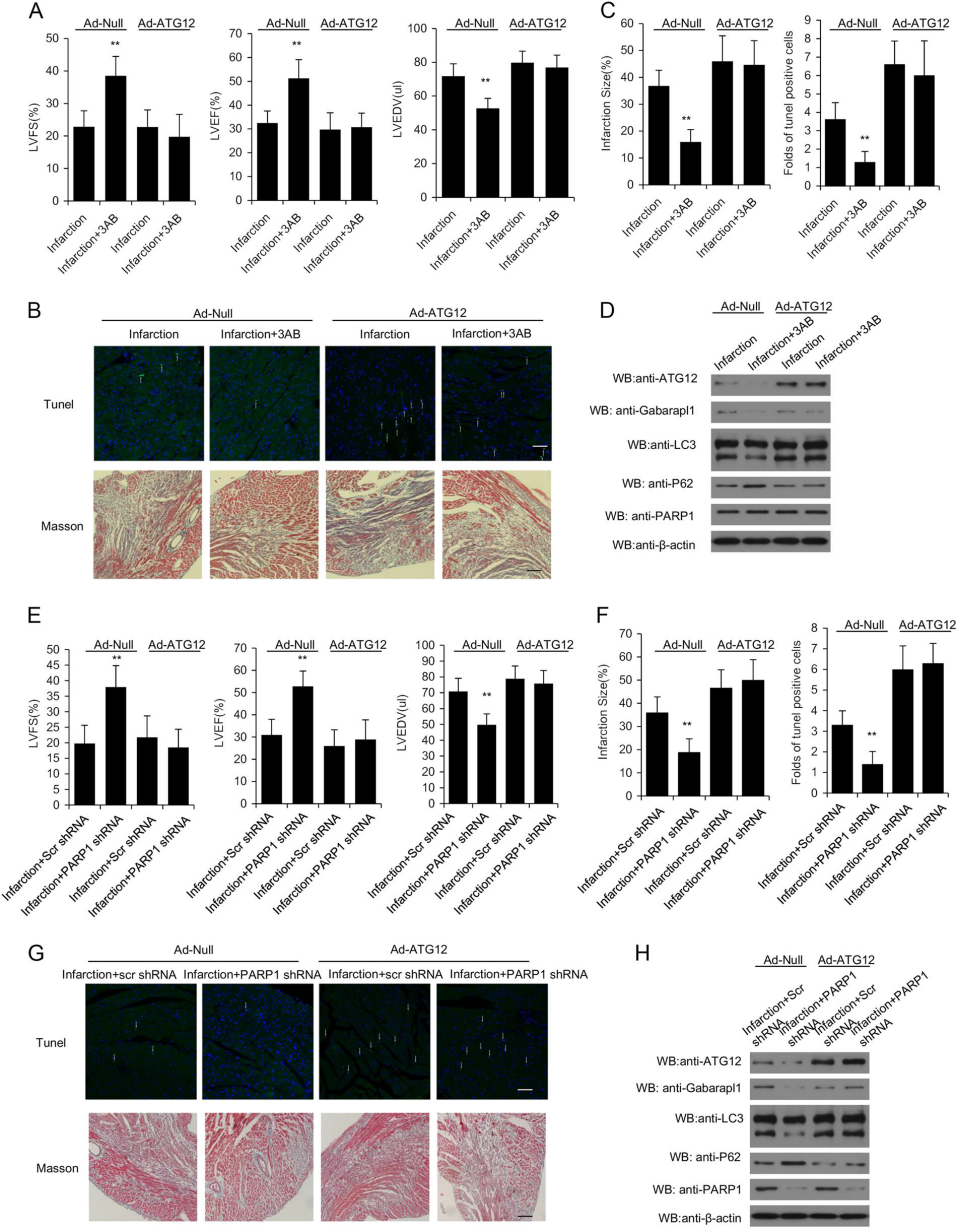
PARP1 inhibition protects the heart against ischemia by inhibition of autophagy initiation. After 8–10-week-old male C57/BL6J mice were subjected to LAD ligation, mice either were intraperitoneal injected with 3AB (30 mg/kg/day) plus intramuscular injected with Ad-Null or Ad-ATG (10 μl (1 × 10^10^ pfu/ml)) (**a**–**d**). After eight- to ten-week-old male C57/BL6J mice were subjected to LAD ligation, mice either were intramuscular injectied with PARP1 shRNA or Scr shRNA plus with Ad-Null or Ad-ATG (10 μl (1 × 10^10^ pfu/ml)) (**e**–**h**). **a** and **e** The quantification of LVFS %, LVEF %, and LVEDV (ul) were conducted. *N* = 5 for each group. ^**^P < 0.01 vs. control; ^##^*P* < 0.01 vs. infarction. The representative TUNEL and Masson staining images were accessed (**b**, **c**, **f**, and **g**). Western-blot assay was used to detect the expression of Gabarapl1, ATG12, LC3, PARP1 and p62 in the areas proximal to infarct/ischemic zone in MI mice (**d** and **h**)

The adenovirus ATG12 (Ad-ATG12) was then injected into MI mice to promote formation of autophagosome. Compared with Ad-null group, echocardiography data demonstrated Ad-ATG12 treatment did not change the LVFS, LVEF and LVEDD of MI heart (Fig. 7a). Furthermore, ATG12 over-expression eliminated the protective effect of 3AB treatment (Fig. 7a). TUNEL and Masson staining presented that over-expression of ATG12 slightly increased the area of myocardial infarction and cardiac apoptosis, and Ad-ATG12 administration abrogated the inhibition effect of 3AB on infarct area and myocardial apoptosis (Fig. 7b, c). Furthermore, western blot also showed that ATG12 administration abolished the regulation of 3AB on cardiac expressions of autophagy related genes LC3, Gabarapl1 and p62 (Fig. 7d). Consistently, overexpression of ATG12 also abrogated the anti-autophagy effect of PARP1 shRNA treatment (Fig. 7e–h).

All these suggested that PARP1 inhibition protected against myocardial ischemia by blocking autophagy initiation.

## Discussion

Autophagy is closely related with cell death and apoptosis in eukaryotic organisms^31^. Although it is proved that PARP1 activation plays a curial role in oxidative stress-induced cell death^32^, the precise understanding of underlying mechanisms remains elusive. Here we report several new and important findings. First, PARP1 is activated in response to stress such as starvation/nutrient deprivation. PARP1 activation promotes expression of autophagic pathway genes in cardiomyocytes. Another key finding of this study is that PARP1-dependent autophagy is detrimental effect for mitochondrial homeostasis and cardiomyocyte apoptosis. Mechanically, PARP1 mediated poly(ADP-ribosyl)ation excludes histone H1, restrains FoxO3a in the nuclei, thus promoting FoxO3a DNA binding and transcriptional activity, resulting into increased autophagy related-gene expression. To our knowledge, this is the first study showing that the inhibition of PARP1 reduces ischemia-induced autophagy and performs its cardio protective effect.

PTM of FoxO3a is a crucial step in FoxO3a transactivation. Poly(ADP-ribosy)lation is an important post-transcriptional modification of proteins^11,33^. It may enhance or inhibit the DNA binding of different nuclear proteins by changing their respective structure. Studies have also reported that the nucleosome binding activity of transcription factors are regulated by poly(ADP-ribosyl)ation, such as PPARα, ERα, FXR^20,34–36^. In this study, we showed that PARP1-induced poly(ADP-ribosy)lation was required for starvation-induced FoxO3a nuclear accumulation through promoting FoxO3a dephosphorylation. The poly(ADP-ribosy)lation of FoxO3a resulted in an overall effect of increasing FoxO3a phosphorylation in the cytosol while decreasing it in the nucleus (Fig. 3). Furthermore, de-phosphorylation of FoxO3a lead to increased DNA binding activity, may therefore contribute to a promotion in transcriptional activity.

Previous study demonstrated the reciprocal relationship between histone H1 and PARP1 for their binding at promoters and other genomic locations^24^. The interplay between histone H1, PARP1 and FoxO3a is also explored in the present study. Our results indicate that PARP1 does not only directly poly(ADP-ribosyl)ate FoxO3a, but also displace the linker histone H1 from nucleosomes by poly(ADP-ribosyl)ating it or by competing for overlapping binding sites on the nucleosomes. Furthermore, starvation/nutrient deprivation facilitates remission of histone H1 from enhancer of FoxO3a target gene promoter, and promotes FoxO3a recruitment to its binding sites on autophagy related gene promoter. According to previous report, poly(ADP-ribosyl)ation probably induces a structural change of PPARα/SIRT1 complex (SIRT1 is key regulator of PPARα), and thus SIRT1 can no longer target poly(ADP-ribosyl)ated PPARα to the target gene promoter, which leads to the down-regulation of PPARα target genes^34^. Hence, we speculate that PARP1 might change the structure conformation of FoxO3a target gene promoter by poly(ADP-ribosyl)ation of Histone H1, thus leading to the exposure of FoxO3a binding site to poly(ADP-ribosyl)ated FoxO3a, triggering the activation of FoxO3a and expression of autophagy related genes.

The role of FoxO3a in autophagy is supported by evidence from studies^7,37^. Accordingly, a recent study has shown that FoxO3 is a trigger for mitochondrial ROS, and that this feedback-loop causes secondary activation of additional FoxO3 molecules leading to the second wave of ROS accumulation and apoptotic cell death^38^. During ischemic injury or starvation, the cellular ATP content decreases and AMP accumulates because of energy depletion. Previous studies also proved that PARP-1 is a positive modulator of starvation-induced autophagy. Under starvation, ROS-induced DNA damage activates PARP-1, leading to ATP depletion^39^. Here, we also show that PARP1 activation promotes the starvation induced-inactivity of basal oxygen consumption and ATP contents in myocardial cells. Mitochondria are organelles that produce ATP and play an important role in cell death and survival. We then concluded that inhibition of PARP1 could recover the impaired mitochondrial function during cardiomyocyte autophagy. After myocardial ischemia injury, damaged mitochondria would activate autophagy observed, which can lead to cell apoptosis and necrosis. Besides, impaired autophagy inhibits the conversion of cardiac fibroblasts to cardiac myofibroblasts and adverse cardiac remodeling^40^. Our previous study proved that inhibition of PARP prevents fibrosis^38,41^. Consistently, we also found that PARP1 inhibition could attenuate autophagy initiation, cell apoptosis and cardiac remolding in MI mice. Thus, we demonstrated that PARP1 is critically involved in the pathogenesis of myocardial ischemia injury through mediating cardiomyocyte autophagy.

In the present study, PARP1 is activated in process of starvation-induced autophagy initiation. Activation of PARP1 facilitates autophagy through poly(ADP-ribosyl)ation of FoxO3a, which impairs mitochondrial metabolism, promotes cardiomyocyte apoptosis, and exacerbates cardiac remolding induced by myocardial ischemia. Moreover, poly(ADP-ribosyl)ation of histone H1 increases recruitment of poly(ADP-ribosyl)ated FoxO3a to the autophagy related gene promoters. These findings highlight the key role of PARP1 activation in the myocardial autophagy. Our data for the first time suggests that inhibition of PARP1 is novel potential strategy against autophagy, might reduce the permanent damage to cardiomyocytes, such as ischemic heart disease or myocardial infarction.

## Electronic supplementary material

supplementary

## References

[1] Gatica D, Chiong M, Lavandero S, Klionsky DJ (2015). Molecular mechanisms of autophagy in the cardiovascular system. Circ. Res..

[2] Lavandero S, Chiong M, Rothermel BA, Hill JA (2015). Autophagy in cardiovascular biology. J. Clin. Invest..

[3] Shirakabe A, Ikeda Y, Sciarretta S, Zablocki DK, Sadoshima J (2016). Aging and autophagy in the heart. Circ. Res..

[4] Eijkelenboom A, Burgering BM (2013). FOXOs: signalling integrators for homeostasis maintenance. Nat. Rev. Mol. Cell Biol..

[5] Tsuchiya K, Ogawa Y (2017). Forkhead box class O family member proteins: the biology and pathophysiological roles in diabetes. J. Diabetes Investig..

[6] Lam EW, Brosens JJ, Gomes AR, Koo CY (2013). Forkhead box proteins: tuning forks for transcriptional harmony. Nat. Rev. Cancer.

[7] Mammucari C (2007). FoxO3 controls autophagy in skeletal muscle in vivo. Cell. Metab..

[8] Xie Q (2012). Lysine methylation of FOXO3 regulates oxidative stress-induced neuronal cell death. EMBO Rep..

[9] Tikhanovich I (2014). Regulation of FOXO3 by phosphorylation and methylation in hepatitis C virus infection and alcohol exposure. Hepatology.

[10] Hu C (2017). hTERT promotes the invasion of gastric cancer cells by enhancing FOXO3a ubiquitination and subsequent ITGB1 upregulation. Gut.

[11] Kim MY, Zhang T, Kraus WL (2005). Poly(ADP-ribosyl)ation by PARP-1: ‘PAR-laying’ NAD+ into a nuclear signal. Genes Dev..

[12] Kim MY, Mauro S, Gevry N, Lis JT, Kraus WL (2004). NAD^+^-dependent modulation of chromatin structure and transcription by nucleosome binding properties of PARP-1. Cell.

[13] Pirrotta V (2004). The ways of PARP. Cell.

[14] Krishnakumar R, Kraus WL (2010). The PARP side of the nucleus: molecular actions, physiological outcomes, and clinical targets. Mol. Cell.

[15] Molnar A (2006). Activation of the poly(ADP-ribose) polymerase pathway in human heart failure. Mol. Med..

[16] Esposito E, Cuzzocrea S (2009). Superoxide, NO, peroxynitrite and PARP in circulatory shock and inflammation. Front. Biosci..

[17] Pacher P, Szabo C (2007). Role of poly(ADP-ribose) polymerase 1 (PARP-1) in cardiovascular diseases: the therapeutic potential of PARP inhibitors. Cardiovasc. Drug. Rev..

[18] Xu S, Bai P, Little PJ, Liu P (2014). Poly(ADP-ribose) polymerase 1 (PARP1) in atherosclerosis: from molecular mechanisms to therapeutic implications. Med. Res. Rev..

[19] Xu Wenjing, Wang Cheng, Liang Minglu, Chen Long, Fu Qin, Zhang Fengxiao, Wang Yan, Huang Dan, Huang Kai (2017). A20 prevents obesity-induced development of cardiac dysfunction. Journal of Molecular Medicine.

[20] Wang C (2013). Poly(ADP-ribose) polymerase 1 promotes oxidative-stress-induced liver cell death via suppressing farnesoid X receptor alpha. Mol. Cell. Biol..

[21] Gao E (2010). A novel and efficient model of coronary artery ligation and myocardial infarction in the mouse. Circ. Res..

[22] Sengupta A, Molkentin JD, Yutzey KE (2009). FoxO transcription factors promote autophagy in cardiomyocytes. J. Biol. Chem..

[23] Eijkelenboom A (2013). Genome-wide analysis of FOXO3 mediated transcription regulation through RNA polymerase II profiling. Mol. Syst. Biol..

[24] Krishnakumar R (2008). Reciprocal binding of PARP-1 and histone H1 at promoters specifies transcriptional outcomes. Science.

[25] Fontan-Lozano A (2010). Histone H1 poly[ADP]-ribosylation regulates the chromatin alterations required for learning consolidation. J. Neurosci..

[26] Rambold AS, Lippincott-Schwartz J (2011). Mechanisms of mitochondria and autophagy crosstalk. Cell Cycle.

[27] Song S, Tan J, Miao Y, Li M, Zhang Q (2017). Crosstalk of autophagy and apoptosis: Involvement of the dual role of autophagy under ER stress. J. Cell. Physiol..

[28] Li Shiguo, Liu Chao, Gu Lei, Wang Lina, Shang Yongliang, Liu Qiong, Wan Junyi, Shi Jian, Wang Fang, Xu Zhiliang, Ji Guangju, Li Wei (2016). Autophagy protects cardiomyocytes from the myocardial ischaemia-reperfusion injury through the clearance of CLP36. Open Biology.

[29] Nakai A (2007). The role of autophagy in cardiomyocytes in the basal state and in response to hemodynamic stress. Nat. Med..

[30] Talman V, Ruskoaho H (2016). Cardiac fibrosis in myocardial infarction-from repair and remodeling to regeneration. Cell Tissue Res..

[31] Yonekawa T, Thorburn A (2013). Autophagy and cell death. Essays Biochem..

[32] Jiang HY (2018). The dual role of poly(ADP-ribose) polymerase-1 in modulating parthanatos and autophagy under oxidative stress in rat cochlear marginal cells of the stria vascularis. Redox Biol..

[33] Andreone TL, O’Connor M, Denenberg A, Hake PW, Zingarelli B (2003). Poly(ADP-ribose) polymerase-1 regulates activation of activator protein-1 in murine fibroblasts. J. Immunol..

[34] Huang K (2017). PARP1-mediated PPARalpha poly(ADP-ribosyl)ation suppresses fatty acid oxidation in non-alcoholic fatty liver disease. J. Hepatol..

[35] Zhang F (2013). Poly(ADP-ribose) polymerase 1 is a key regulator of estrogen receptor alpha-dependent gene transcription. J. Biol. Chem..

[36] Huang D, Yang C, Wang Y, Liao Y, Huang K (2009). PARP-1 suppresses adiponectin expression through poly(ADP-ribosyl)ation of PPAR gamma in cardiac fibroblasts. Cardiovasc. Res..

[37] Schips TG (2011). FoxO3 induces reversible cardiac atrophy and autophagy in a transgenic mouse model. Cardiovasc. Res..

[38] Hagenbuchner J, Ausserlechner MJ (2013). Mitochondria and FOXO3: breath or die. Front. Physiol..

[39] Rodriguez-Vargas JM (2012). ROS-induced DNA damage and PARP-1 are required for optimal induction of starvation-induced autophagy. Cell Res..

[40] Gupta SS (2016). Inhibition of autophagy inhibits the conversion of cardiac fibroblasts to cardiac myofibroblasts. Oncotarget.

[41] Wang Y (2013). Inhibition of PARP prevents angiotensin II-induced aortic fibrosis in rats. Int. J. Cardiol..

