# Supplementary material for: PARP1 promote autophagy in cardiomyocytes via modulating FoxO3a transcription
Source: Cell Death Dis. 2018 Oct 15;9(11):1047. doi: 10.1038/s41419-018-1108-6 (PMC6189197; doi:10.1038/s41419-018-1108-6)
Supplement: Supplementary file 1 — supplementary [file 41419_2018_1108_MOESM1_ESM.docx]

**Supplementary Figures**

**
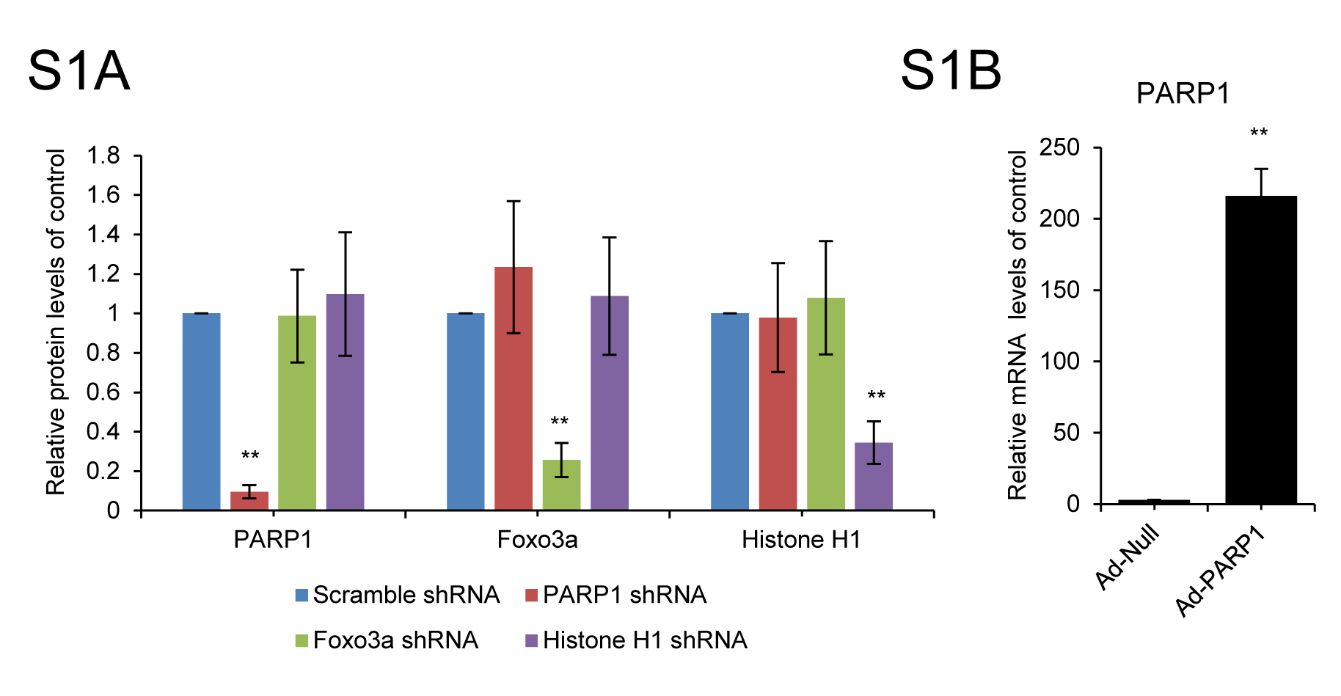
**

**Supplementary Figure 1** The relative mRNA level of PARP1, FoxO3a, Histone H1 were determined in different shRNA lentivirus **(S1A)** or overexpressed adenovirus **(S1B)** infected primary rat cardiomyocytes. N=3. ^**^P<0.01 vs. control.

**
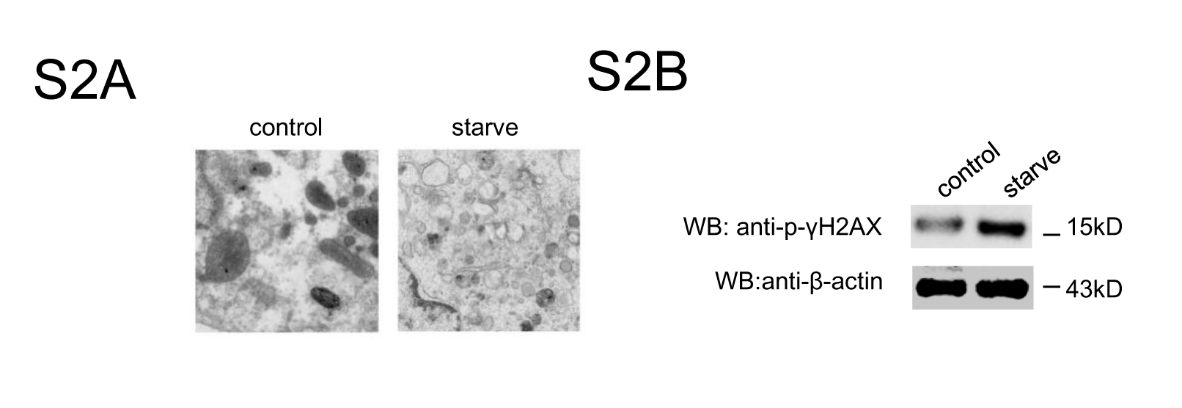
**

**Supplementary Figure 2** Primary rat cardiomyocytes were starved for 24h. (**S2A**) Representative electron microscopy are displayed to assess the effect on starvation-induced autophagy. (**S2B**) The protein level of DNA damage marker (γ-H2AX) were detected by western blot analysis.


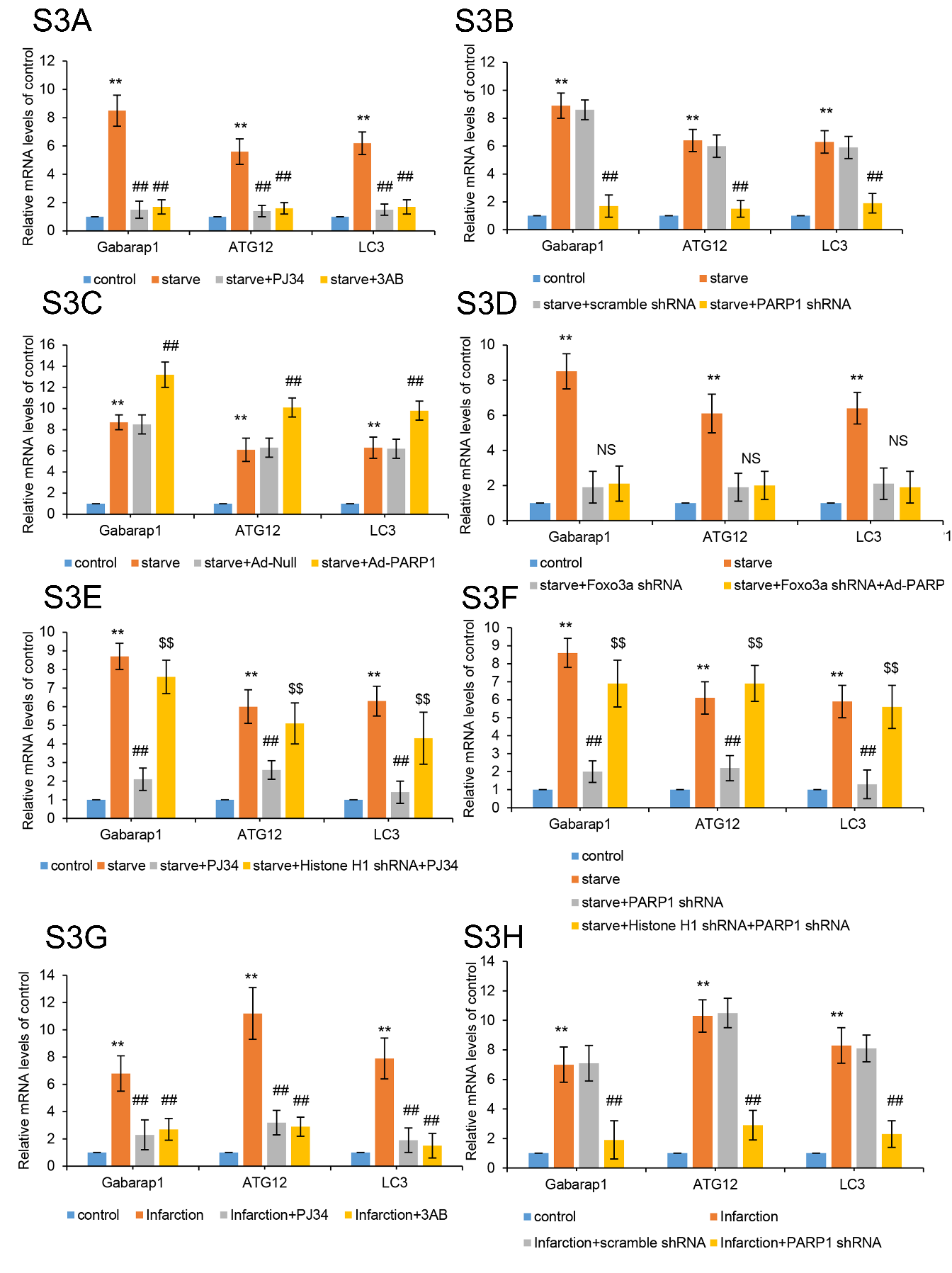


**Supplementary Figure 3** (**S3A**-**S3F**) The relative mRNA expression of Gabarapl1, ATG12 and LC3 in NRCMs under indicated treatment. N=5. ^**^P<0.01 vs. control; ^##^P<0.01 vs. Scr shRNA or Ad-Null under starve; ^$$^P<0.01 vs. PJ34, PARP shRNA or Ad-Null under starve. (**S3G** and **S3H**) The relative mRNA expression of Gabarapl1, ATG12 and LC3 in heart under indicated treatment. N=5. ^**^P<0.01 vs. control; ^##^P<0.01 vs. Scr shRNA or Ad-Null under infarction.

**
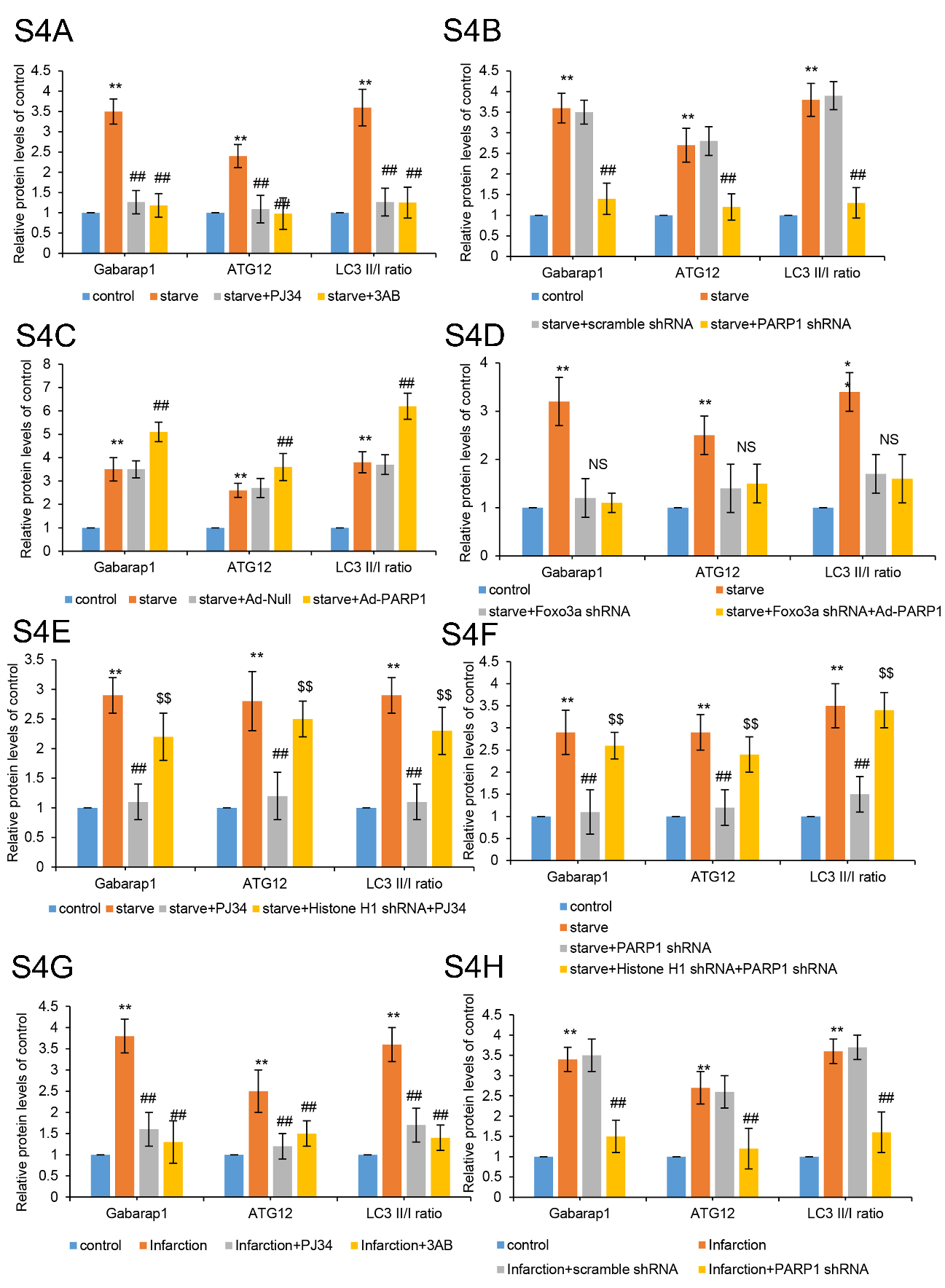
**

**Supplementary Figure 4 The related densitometry data, n-numbers and relevant statistics were provided.** (**S4A**-**S4F**) The relative protein expression of Gabarapl1, ATG12 and LC3 in NRCMs under indicated treatment. N=5. ^**^P<0.01 vs. control; ^##^P<0.01 vs. Scr shRNA or Ad-Null under starve; ^$$^P<0.01 vs. PJ34, PARP shRNA or Ad-Null under starve. (**S4G** and **S4H**) The relative protein expression of Gabarapl1, ATG12 and LC3 in heart under indicated treatment. N=5. ^**^P<0.01 vs. control; ^##^P<0.01 vs. Scr shRNA or Ad-Null under infarction.


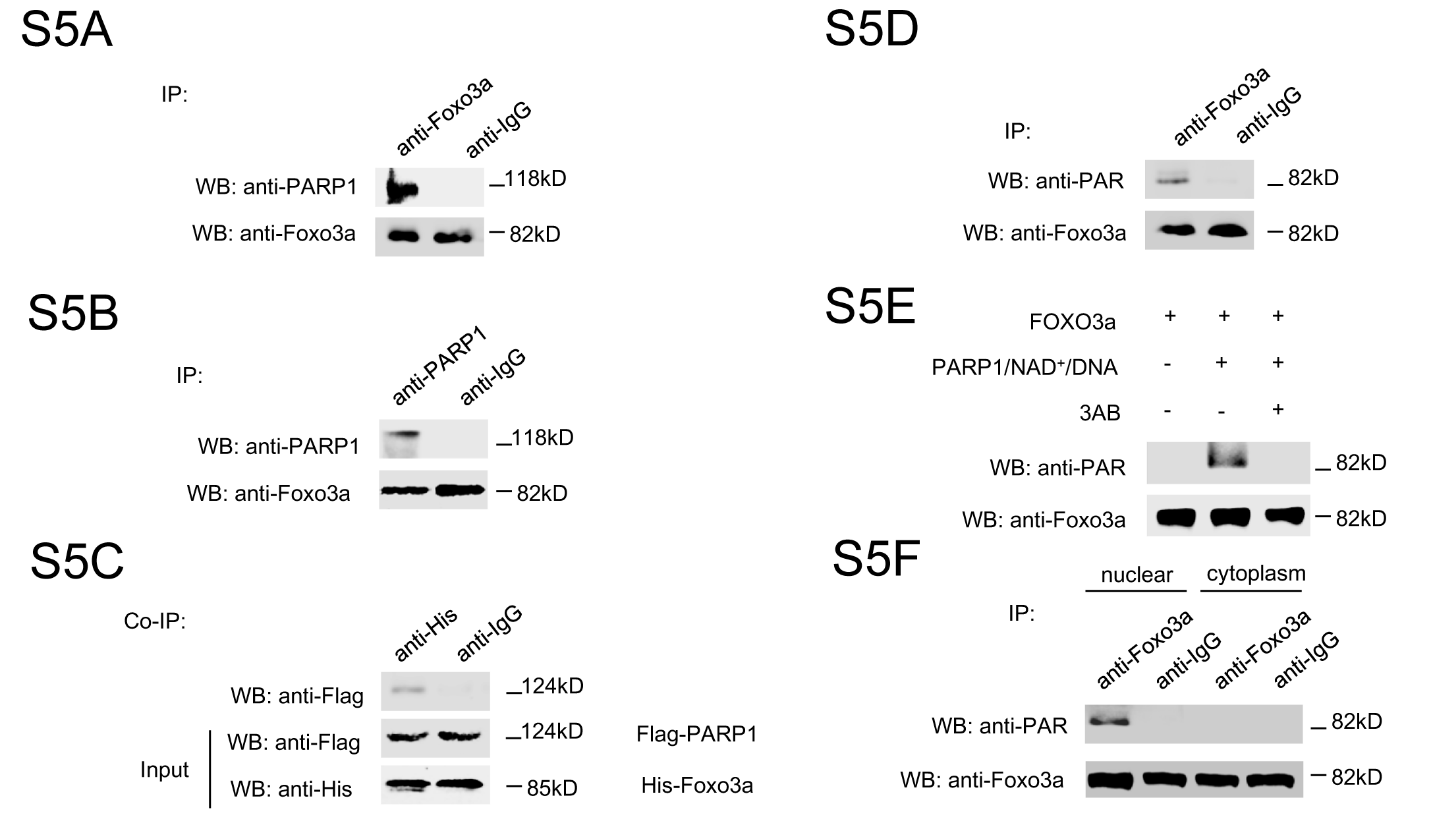


**Supplementary Figure 5** (**S5A** and **S5B**) Coimmunoprecipitation assays of FoxO3a-bound proteins from Primary neonatal rat cardiomyocytes, followed by Western blot assays using an anti-PARP1 antibody and vice versa. Nonspecific IgG served as a negative control. (**S5C**) Primary neonatal rat cardiomyocytes were transfected with His-tagged FoxO3a and Flag-tagged PARP1, coimmunoprecipitation assays demonstrated the specific binding of PARP1 to FoxO3a, IgG served as a negative control. (**S5D**) Primary neonatal rat cardiomyocytes were subjected to an immunoprecipitation assay with an anti-FoxO3a antibody, followed by a Western blot assay using an anti-PAR antibody. (**S5E**) Recombinant FoxO3a proteins were incubated either with a vehicle (PBS), with PARP1, NAD+, and active DNA, or with PARP1, NAD^+^, active DNA, and 3AB, as indicated. Western blot assays were used to detect the poly(ADP-ribosyl)ation levels of FoxO3a. (**S5F**) Nuclear extracts and cytoplasm extracts from Primary neonatal rat cardiomyocytes were subjected to an immunoprecipitation assay with an anti-FoxO3a antibody, followed by a Western blot assay using an anti-PAR antibody.


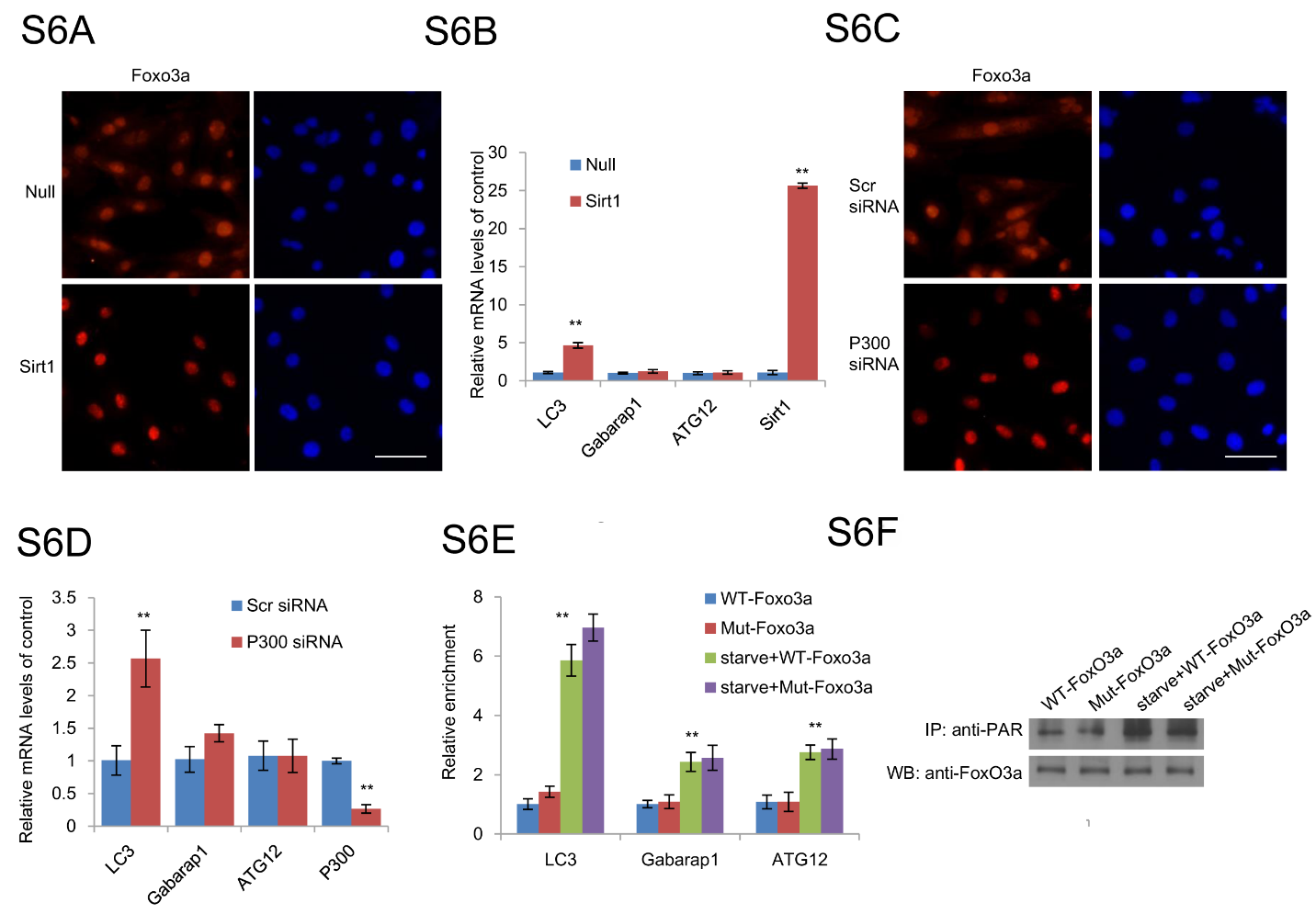


**Supplementary Figure 6** Primary rat cardiomyocytes were transfected with Null or Sirt1 plasmids for 24 h followed by starvation (24h). (**S6A**) Confocal immunofluorescence assay was used to detect the expression of FoxO3a (red fluorescence), Hoechst (blue fluorescence) was used to stain the cell nuclei (scale bar=40 µm). (**S6B**) The relative mRNA expression of Gabarapl1, ATG12 and LC3 were determined by Realtime qPCR. N=5. ^**^P<0.01 vs. control; Primary rat cardiomyocytes were transfected with Null or Sirt1 plasmids for 24 h followed by starvation (24h). (**S6C**) Confocal immunofluorescence assay was used to detect the expression of FoxO3a (red fluorescence), Hoechst (blue fluorescence) was used to stain the cell nuclei (scale bar=40 µm). (**S6D**) The relative mRNA expression of Gabarapl1, ATG12 and LC3 were determined by Realtime qPCR. N=5. ^**^P<0.01 vs. control; (**S6E** **and S6F**) Primary neonatal rat cardiomyocytes transfected with WT-FoxO3a Mut-FoxO3a for 24h, and then treated with or without starvation (24h). Soluble chromatin was then prepared for ChIP assays with antibodies against the Flag (**S6E**). Coimmunoprecipitation assays were used to detect the poly(ADP-ribosyl)ation levels of FoxO3a (**S6F**).


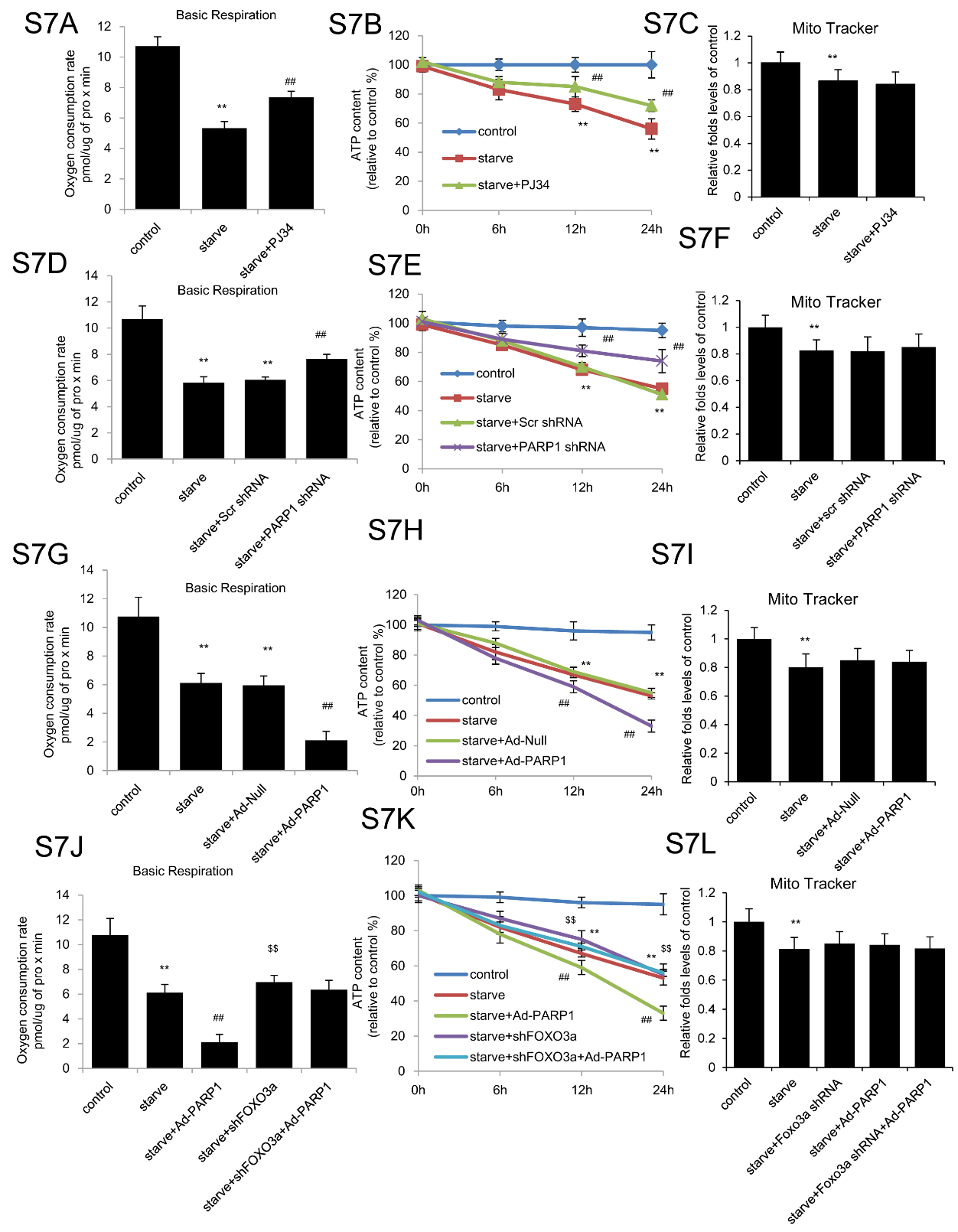


**Supplementary Figure 7**. (**S7A-S7C**) Primary rat cardiomyocytes were treated with PJ34 (10 μM) and starved for 24 hours. Oxygen consumption was measured in cardiomyocytes by Clark electrode (**A**). Intracellular ATP levels were determined by luminescence using the luciferin/luciferase assay (**B**). Mitochondria content was analysed by flow cytometer using MitoTracker Dye. (**S7D-S7F**) Cells were infected with PARP1 shRNA or Scr shRNA for 24h and then starved for 24h. Oxygen consumption(**D**), intracellular ATP levels(**E**) and Mitochondria content (**F**) were detected. (**S7G-S7I**) Cells were infected with Ad-PARP1 or Ad-Null before starvation for 24h. Oxygen consumption (**G**), intracellular ATP levels (**H**) and Mitochondria content (**I**) were detected. (**S7J-S7L**) Cells were infected with FoxO3a shRNA or FoxO3a shRNA+Ad-PARP1 as indicated before starvation for 24h. Oxygen consumption (**J**), intracellular ATP levels (**K**) and Mitochondria content (**L**) were detected. ^**^P < 0.01 vs. control; ^##^P < 0.01 vs. starve or starve+scramble shRNA or starve+Ad-Null; ^$$^P < 0.01 vs. starve+Ad-PARP1


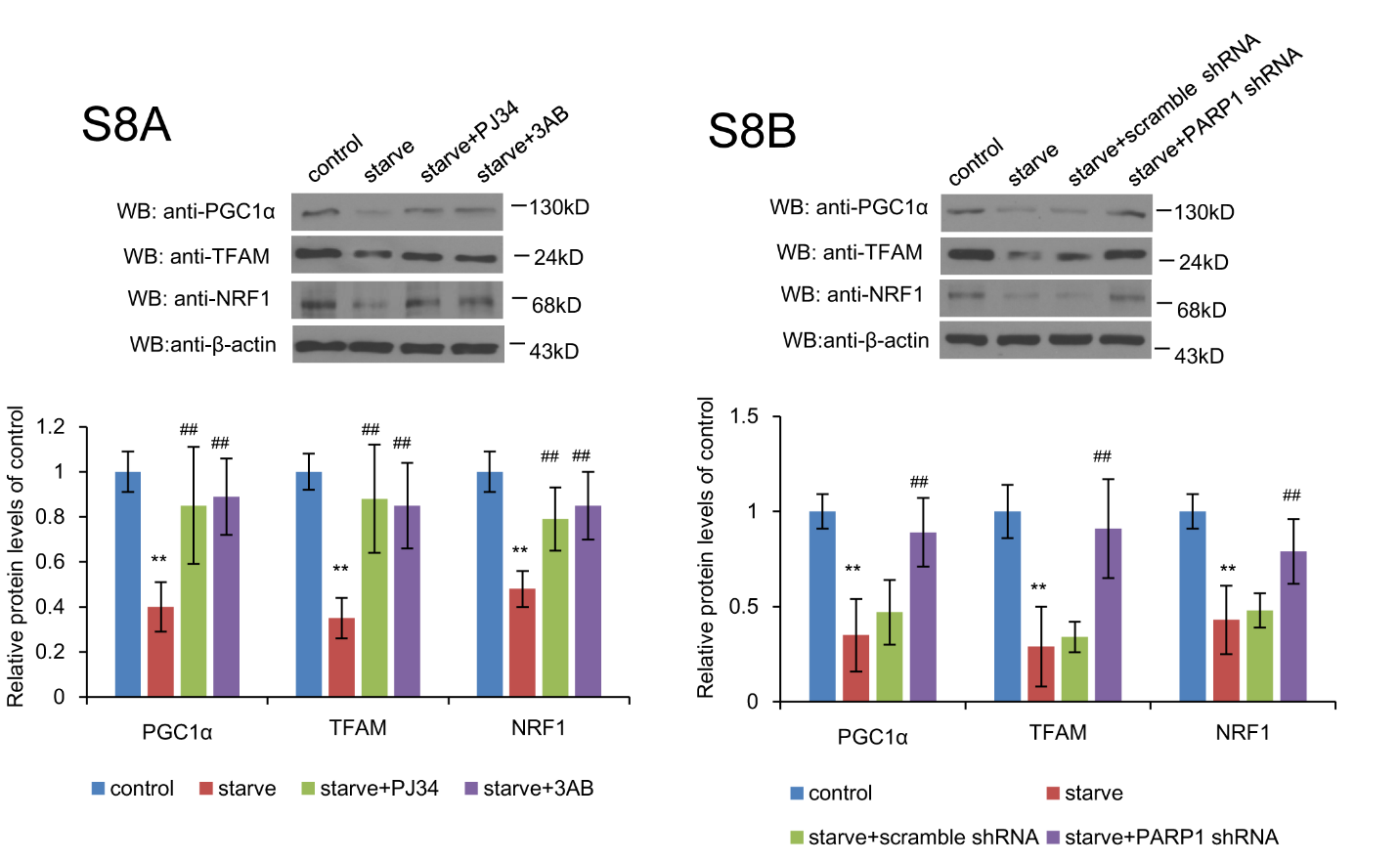


**Supplementary Figure 8**. Primary neonatal rat cardiomyocytes were pre-treated with 3AB (10 mM) or PJ34 (10 µM), or transfected with PARP1 shRNA or an unrelated shRNA for 24h followed by starvation (24h). The protein level of PGC1α, TFAM and NRF1 were detected by western blot analysis. N=5. ^**^P<0.01 vs. control; ^##^P<0.01 vs. starve or Scr shRNA under starve.


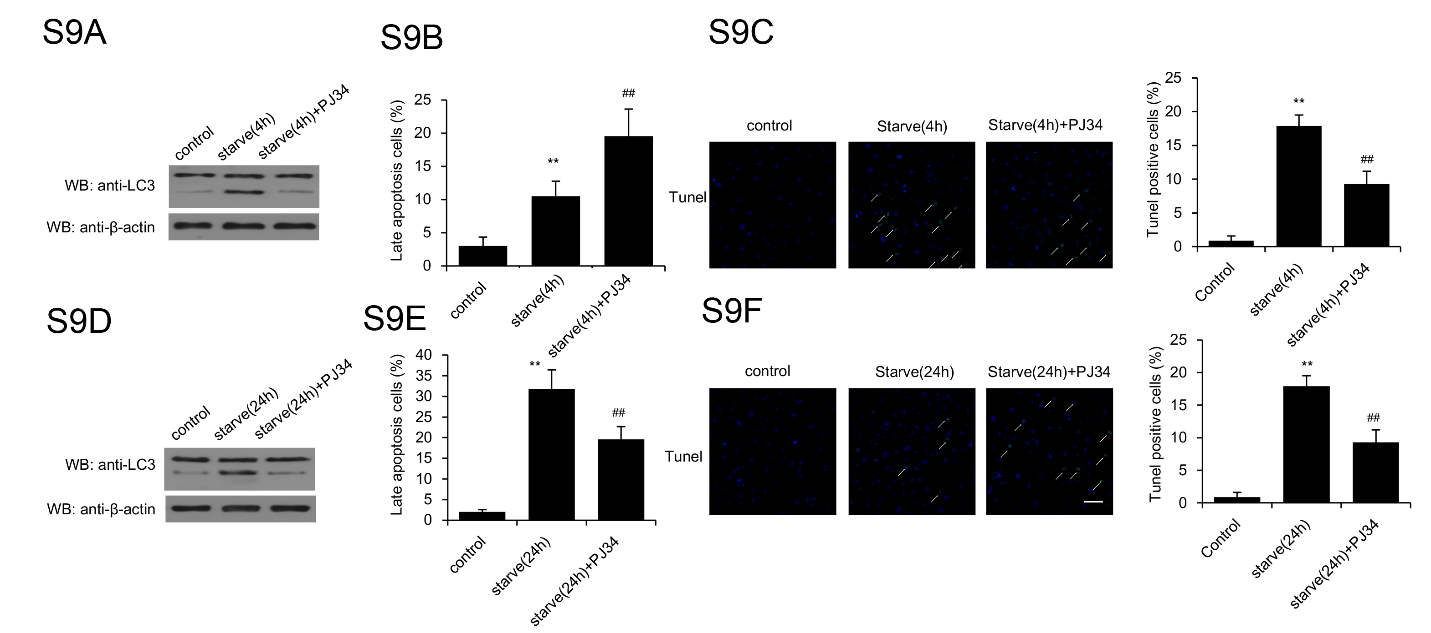


**Supplementary Figure 9** Primary rat cardiomyocytes were treated with PJ34 (10 μM) and starved for 4 hours or 24 hours. The protein level of LC3 were determined by Western blot assays (**S9A and S9D**). PI/Annexin V-labelled apoptosis cells were analysed by flow cytometer (**S9B and S9E**). The representative TUNEL staining were accessed (**S9C and S9F**). N=5. ^**^P<0.01 vs. control; ^##^P<0.01 vs. starve.


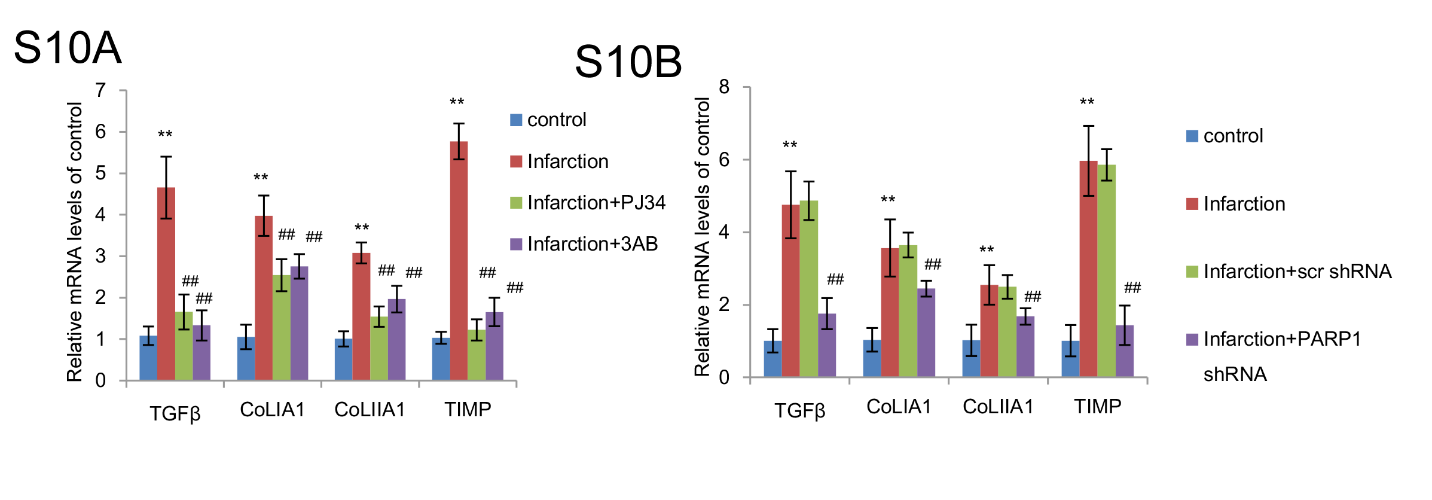


**Supplementary Figure 10** After eight- to ten-week-old male C57/BL6J mice were subjected to LAD ligation, mice either were intraperitoneal injected with PJ34 (20 mg/kg/day) or 3AB (30 mg/kg/day) or intramuscular injected with PARP1 shRNA or an unrelated shRNA. Realtime qPCR assay was used to detect the expression of TGFβ, ColIA1, ColIIA1 and TIMP in the areas proximal to infarct/ischemic zone in MI mice. N = 5 for each group. ^**^P < 0.01 vs. control; ^##^P < 0.01 vs. infarction.


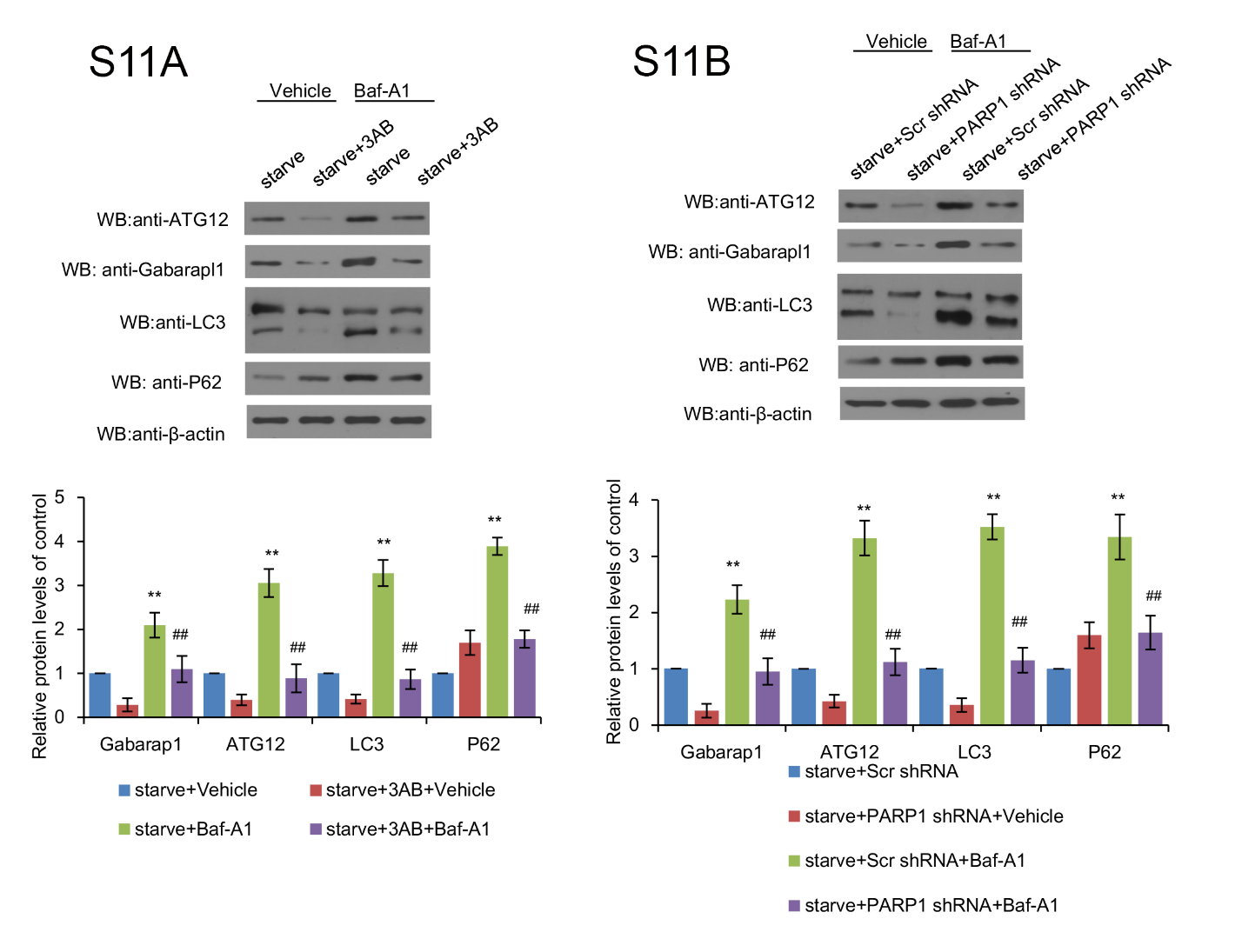


**Supplementary Figure 11** PJ34 (10 µM) pretreated or PARP1 shRNA infected primary rat cardiomyocytes were treated with Baf A1 (100 nM) and starved for 24 hours. Western blot assays were used to test the expression of autophagy marker (Gabarapl1, ATG12, LC3), PARP1 and p62. N=5. ^**^P<0.01 vs. control; ^##^P<0.01 vs. starve. N=5. **P<0.01 vs. control; ^##^P<0.01 vs Baf A1 under starve.
